# The orbitofrontal cortex maps future navigational goals

**DOI:** 10.1038/s41586-021-04042-9

**Published:** 2021-10-27

**Authors:** Raunak Basu, Robert Gebauer, Tim Herfurth, Simon Kolb, Zahra Golipour, Tatjana Tchumatchenko, Hiroshi T. Ito

**Affiliations:** 1 Max Planck Institute for Brain Research, Frankfurt am Main, Germany; 2 Institute of Experimental Epileptology and Cognition Research, Life and Brain Center, Universitätsklinikum Bonn, Bonn, Germany

**Keywords:** Spatial memory, Neural circuits

## Abstract

Accurate navigation to a desired goal requires consecutive estimates of spatial relationships between the current position and future destination throughout the journey. Although neurons in the hippocampal formation can represent the position of an animal as well as its nearby trajectories^1–7^, their role in determining the destination of the animal has been questioned^8,9^. It is, thus, unclear whether the brain can possess a precise estimate of target location during active environmental exploration. Here we describe neurons in the rat orbitofrontal cortex (OFC) that form spatial representations persistently pointing to the subsequent goal destination of an animal throughout navigation. This destination coding emerges before the onset of navigation, without direct sensory access to a distal goal, and even predicts the incorrect destination of an animal at the beginning of an error trial. Goal representations in the OFC are maintained by destination-specific neural ensemble dynamics, and their brief perturbation at the onset of a journey led to a navigational error. These findings suggest that the OFC is part of the internal goal map of the brain, enabling animals to navigate precisely to a chosen destination that is beyond the range of sensory perception.

## Abstract (web summary)

Dedicated cells in the hippocampus and entorhinal cortex map an animal s instantaneous position in space; by contrast, its future goal location is represented in the orbitofrontal cortex, a structure within the broader circuit.

## Main

**Fig. 1:**
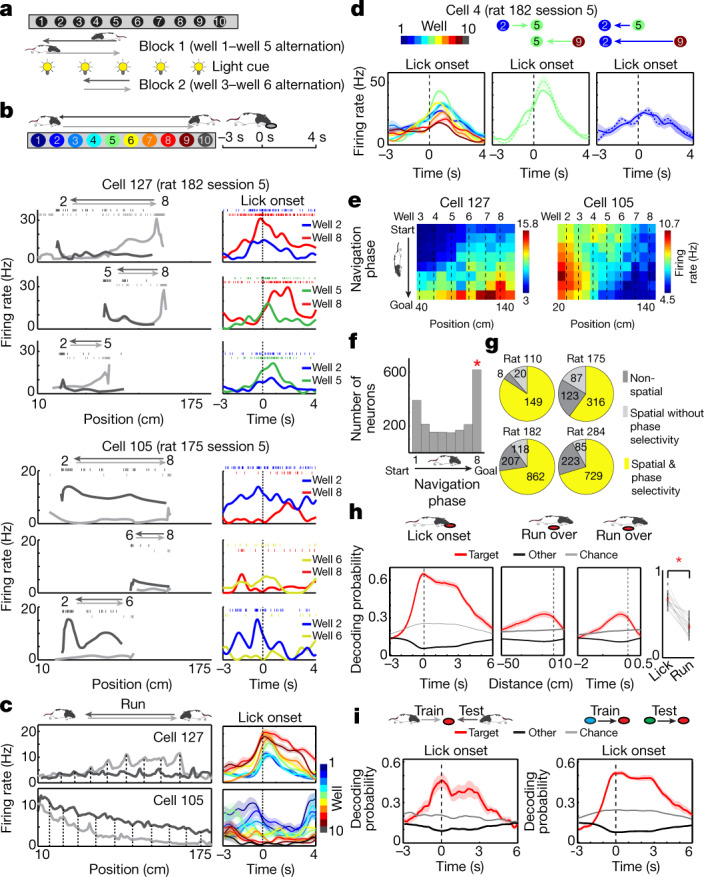
Goal-specific firing of OFC neurons. **a**, Schematic of the task. **b**, Firing of two representative neurons showing spike rasters and rates over position with two running directions in black and grey (left) or over time from lick onset (right). **c**, Firing across trial blocks during running (speed >10 cm s^−1^) or licking. **d**, Left, well-dependent firing across trial blocks. Middle and right, invariant firing to the difference in running direction (middle) or starting well (right). **e**, Colour-coded rate plots along the position and navigation phase of the animal. Shown are all rewarded wells approached in the direction of the higher activity of the neuron. **f**, Distribution of peak firing along navigation phase for all OFC neurons encoding position and navigation phase. *Outlier by generalized extreme studentized deviate test (above the threshold of 607.22 neurons at *P* = 0.05). **g**, Pie charts showing the numbers of neurons with spatial and/or navigation phase tuning. **h**, Left, decoding of licking well (target) and other wells (other). Middle, decoding of run-over well against distance (left) and time (right). Right, comparison of decoding between licking and run-over well, showing individual 18 sessions (grey) and means (red). *P = 1.96 × 10^−4^ in two-sided Wilcoxon signed-rank test. **i**, Decoding of licking well when the corresponding approach direction was excluded from the training of the decoder (left) or when all trials from the corresponding goal well pair were excluded from the training of the decoder (right). In **c**, **d**, **h** and **i**, plots show mean (line) ± s.e.m. (shaded).

**Extended Data Fig. 1:**
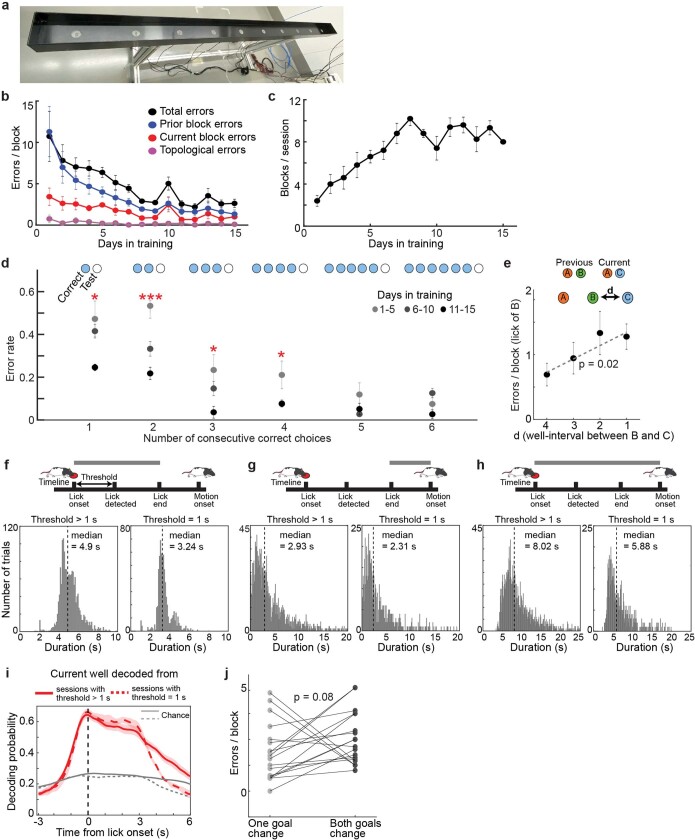
Behavioural performance in the navigation task. **a**, Top view photo of the linear maze used in this study. **b**, Mean number of errors committed per trial block during the first 15 days of training. Prior block errors are defined as incorrect licking of wells that were rewarded in the previous block. Current block errors are defined as incorrect licks of the same well from which the animal obtains its most recent reward in the block (by failing to visit its paired well). Topological errors comprise incorrect licks of the wells immediately next to the correct target well (but if this erroneously licked well was rewarded in the previous block, it was classified as prior block error.) The near absence of topological errors implies that animals form a robust spatial map that enables accurate estimation of well positions. **c**, Mean number of successfully completed blocks per session. As the animals learned the task, block transitions occurred quicker, resulting in a steady increase in the number of successfully completed blocks before saturating after 8 days of training. In panels **b-c**, n = 5 rats for days 1–12, 4 rats for day 13, and 3 rats for days 14-15. **d**, Average error rates after consecutive correct licks. Numbers in the horizontal axis indicate the number of consecutive correct licks prior to the trial being evaluated (as shown in the schematic on top where blue circles denote correct trials and white circles represent the trial whose outcome was analysed. Data are plotted separately for different stages of training. After five and six consecutive correct trials, the probability of making an error in the subsequent trial was reduced to 6.86% and 8.32%, respectively, and was not significantly different across different stages of training (p = 0.0261, 0.0006, 0.0416, 0.0476, 0.2074, and 0.0911 in 1-way ANOVA for error rates following 1-6 consecutive correct trials, respectively; n = 5 rats). We thus introduced a block change of well combinations only after the animal made at least six consecutive correct trials. **e**, Prior block errors were not purely due to the animal’s habitual behaviour. We analysed trials in which the same well was rewarded in both previous and current blocks (well A in the scheme) and its paired well in the previous block (well B in the scheme) was in the middle of journey toward the other goal well in the current block (well C in the scheme). The average number of prior block errors in the trained animals proportionally increased as the distance between well B and well C reduced. Dotted line represents the best fit linear regression line (slope: 0.206, p = 0.02 from two sided t-statistic with the null hypothesis of zero slope. n = 13, 9, 6, 12 sessions from 5 rats for d = 4, 3, 2, 1, respectively). **f-h**, Plots showing the distributions of (f) lick durations, (g) time latencies between the end of licking and the onset of motion, and (h) entire times from lick onset to motion onset. Each distribution was further divided into two plots according to the length of lick threshold. The left plots are based on 12 sessions with the lick threshold of 2 s as well as 1 session with the threshold of 1.5 s, and the right plots are on 5 sessions with the threshold of 1 s. Dotted lines represent the medians. **i**, Decoding probability of the well that was approached and licked by the animal (as in Figure 1h) plotted according to different levels of lick threshold (n = 13 and 5 for the threshold of > 1 s and = 1 s, respectively). Shown are means (line) ± s.e.m (shaded). **j**, No significant difference in error rates between the two consecutive blocks with a common goal well and those without it. Each dot represents the mean from an individual session (p = 0.08 in two-sided Wilcoxon signed-rank test; n = 18 sessions from 4 rats). Error bars in panels **c**, **d**, and **e** denote s.e.m.

**Extended Data Fig. 2:**
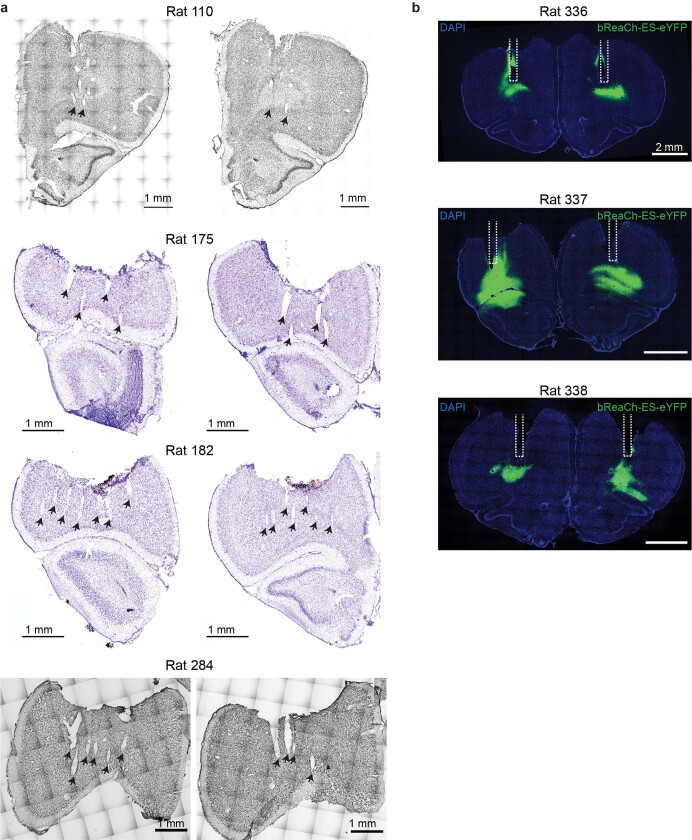
Tetrode locations and bReaCh-ES-eYFP expression in OFC. **a**, Nissl-stained coronal sections of all animals recorded from OFC (4 rats) with tetrode tracks marked with arrows. **b**, Coronal sections showing the expression of bReaCh-ES-eYFP (green) in bilateral OFC of the three animals used for optogenetic perturbation experiments. Dotted white lines indicate the positions of the optic fibres.

We trained five rats on a 2-m-long linear maze with ten water delivery sites or wells (Fig. 1a, Extended Data Fig. 1). The rats were required to visit and lick two given wells alternately to obtain water rewards. The licking of the animal was detected by infrared sensors on individual wells, and water was delivered after the correct well was licked for a fixed amount of time (1 s, 1.5 s or 2 s, consistent across trials in a session). After at least six consecutive correct choices, a new pair of wells started to deliver water, enforcing the updating of goal locations. The rats learned this task over 2 weeks. We implanted a tetrode drive into the ventral and lateral parts of the OFC (Extended Data Fig. 2) and collected data from four rats across 18 sessions, each of which comprised 68–328 simultaneously recorded OFC neurons.

**Extended Data Fig. 3:**
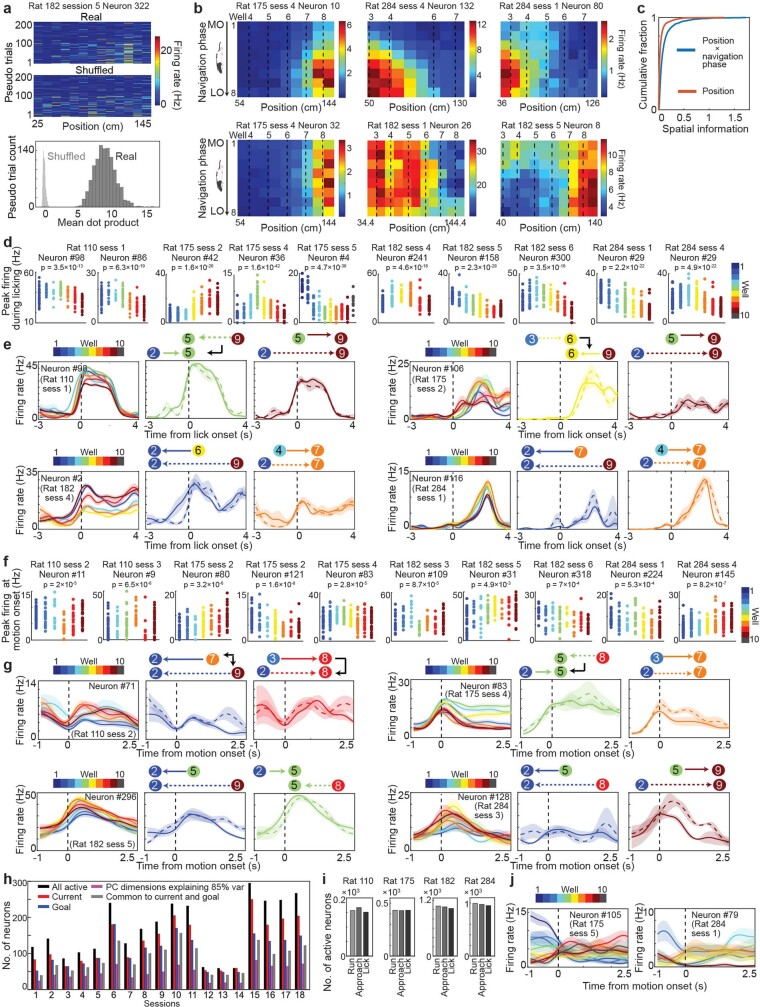
Firing properties of individual OFC neurons. **a**, Procedure for quantifying spatial selectivity (see Methods) of OFC neurons. Colour coded firing rates during 200 pseudotrials and their shuffled counterparts are shown in top and middle panels, respectively. First 100 pseudotrials are during stationary periods (speed < 10 cm/s) and the next 100 are during periods of motion (speed > 10 cm/s). Panel on bottom shows the distributions of mean spatial correlations obtained from 1000 original (light grey) and shuffled (dark grey) sets of pseudotrials for this neuron. **b**, Top: three representative neurons that conjunctively encode spatial location and navigation phase. Bottom: three examples of spatially selective neurons that were not influenced by navigation phases. Same convention as in Figure 1e. **c**, Cumulative frequency of spatial information calculated over firing rates in a 2D space of position × navigation phase (as in **b**) versus that taking into account only positional differences (and hence averaged across phases) for all 2056 neurons representing position and navigation phase conjunctively. Spatial information in the conjunctive position × phase space is greater than the one considering positional differences only (p = 1.78 × 10^−180^ in two-sided Wilcoxon ranksum test). **d**, Peak firing rate of 10 representative neurons during licking. Single dots represent individual trials, and the well identity is colour coded. p-values calculated using one-way ANOVA. **e**, Well-specific but paired-well-independent firing rate of four representative neurons (one from each animal). Same convention as in Figure 1d. **f**, Peak firing rate of 10 representative neurons during −0.5 to 0.5 seconds relative to motion onset. Single dots represent individual trials and are coloured based on the identity of goal well. p-values calculated using one-way ANOVA. **g**, Four representative neurons with goal-well dependent but start-well independent firing at motion onset. p-values calculated using one-way ANOVA. **h**, Session-based summary of the numbers of neurons categorized as active (average firing rate > 0.5), current-well selective, and goal-well selectivite (see Methods), together with the number of dimensions explaining 85% of the variance of goal-selective neurons (obtained using PCA). **i**, Total numbers of active neurons (average firing rate > 0.5 Hz) during each of the following behavioural phases; running, approach (duration of 500 ms prior to lick onset), and well-licking. **j**, Firing rate plots of the same representative neurons as in Figure 2a with trials averaged (and coloured) based on ‘current’ well. In panels **e** and **g**, plots show means (line) ± s.e.m. (shaded).

**Extended Data Fig. 4:**
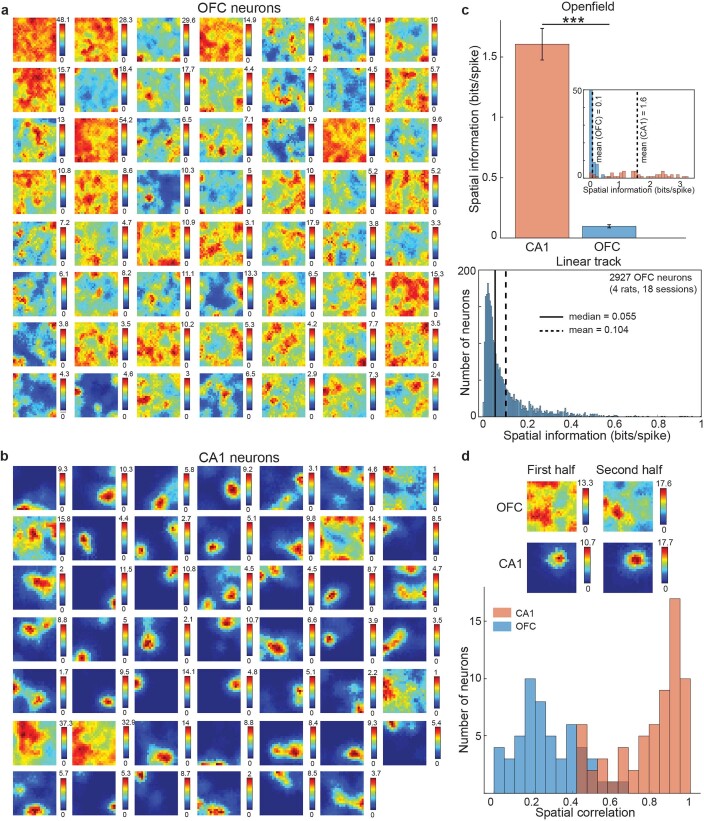
Low spatial information in OFC neurons. **a-b**, Colour-coded firing rate plots of representative OFC neurons and CA1 neurons during a random foraging task in an open-field arena. **c**, Top: spatial information of individual OFC and CA1 neurons in the random foraging task. Error bars denote s.e.m. Inset shows the distribution of spatial information of the OFC and CA1 neurons. ***p = 5.76 × 10^−20^, z = 9.14 in two-sided Wilcoxon rank-sum test. Bottom: distribution of spatial information of individual OFC neurons during the goal-directed navigation task on the linear maze. Solid and dashed vertical lines indicate the median and mean, respectively. **d**, Stability of spatial tuning during the session. Top: rate maps of two representative neurons, each from OFC and CA1, during the first and second halves of the session. Bottom: histogram of spatial correlations of 70 OFC neurons and 65 CA1 neurons. For each neuron, rate maps were calculated separately for the first and second halves of the foraging sessions, and the correlation between the two position-dependent firing-rate vectors was evaluated (spatial correlation: OFC, 0.19 ± 0.02, CA1, 0.79 ± 0.02; two-sided Wilcoxon rank-sum test: z = 9.72, p = 2.45 × 10^−22^).

We found that most OFC neurons increased their spiking as the animal approached the goal well, discriminating its location by changing firing rates (Fig. 1b–d). These neurons, however, showed less position-specific firing to the wells that the animal ran over during navigation. These observations were confirmed by plotting firing rates along maze position conjunctively with navigation phase (defined as positional fraction of journey; Fig. 1e–g). As a population, 80.8% of OFC neurons (2,366 of 2,927) exhibited some degree of spatial tuning on the maze (*z* > 2.57 in spatial correlations compared to shuffled activity), but, in most them (86.9%, 2,056 of 2,366), the spatial tuning was also dependent on navigation phase (*P* < 0.05 in spatial correlations compared to activity shuffled across different navigation phases; Extended Data Fig. 3). We further found that, during a random foraging task in an open-field arena, OFC neurons conveyed significantly lower spatial information than neurons in area CA1 of the hippocampus (Extended Data Fig. 4). These results together suggest that most OFC neurons exhibit location-selective firing in conjunction with the demand and phase of goal-directed journey.

**Extended Data Fig. 5:**
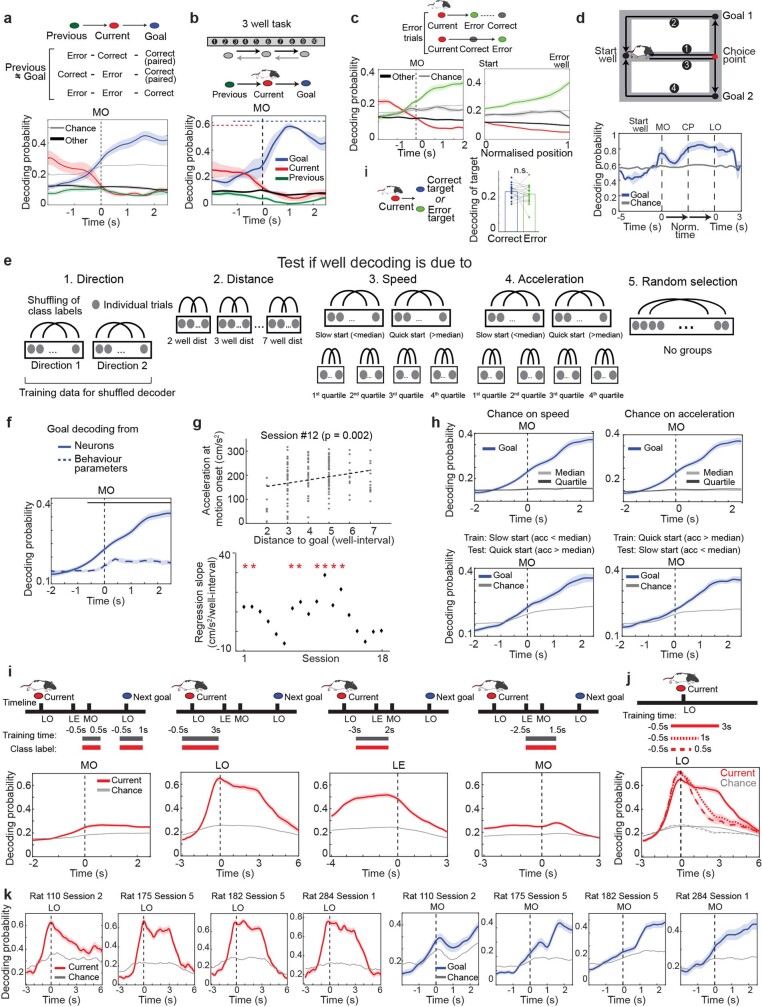
Validation of goal-well decoding. **a**, Decoding of goal well (blue), current well (red), and previous well (green), in the trials where the animal’s next goal and previously-visited well were different due to error trials. n = 18 sessions. MO: motion onset. **b**, Decoding of the wells during a 3-well task. Bottom plot shows the decoding probabilities of the wells when the animal’s next goal and the previous goal were different. The decoder indicated the animal’s next destination but not the previous goal. **c**, Top: decoding on error trials showing the probabilities of current well (red), the animal’s next destination visited incorrectly (green), and the correct well according to the task rule (grey), plotted over time (left) or along positional fraction (right). Bottom: decoding of the animal’s next destination at motion onset between correct (blue) and error (green) trials. Dots represent individual 18 sessions. p = 0.372 in two-sided Wilcoxon signed-rank test. **d**, Top: schematic of the experimental setup and the behaviour paradigm of a continuous alternation task. The correct destination of individual trials switched alternately between Goal 1 and Goal 2. For successful task performance, rats needed to follow the sequence of trajectories outlined by numbers 1 to 4. Two rats were trained with the same strategy as described before^7^, and the performance of both rats reached over 95% accuracy. Bottom: decoding probability of goal well during navigation. Decoding was performed by using a decoder based on quadratic-discriminant analysis that was trained on OFC neural activity during the concatenated time range from −1 s to 1 s relative to motion onset (at the start well) as well as from −2 s to 2 s relative to lick onset (at the goal well). Decoding was restricted to correct trials with trajectory paths 1 and 3 in the top schematic. Each trial was decoded in a leave-one-out cross-validated manner. Decoding performance is plotted across four contiguous time phases: 1) 5 s duration prior to motion onset at the start well, 2) from motion onset to the choice point, 3) from the choice point to lick onset at the goal well, and 4) 3 s duration after lick onset. Due to trial-by-trial variability in the animal’s behaviours, the second and third phases are plotted in normalized time for each. Grey line denotes aggregate chance level from well-based and speed-based null hypotheses (see Methods; chance levels for goal distance and direction were not considered because they were identical between the two goal-directed navigations in the maze). The decoding probability of goal well was significantly greater than chance starting from 0.6 s prior to motion onset until 2.6 s after lick onset at the goal well (decoding probability at motion onset: 0.74 ± 0.06, compared to its chance level of 0.58; n = 4 sessions from 2 rats). MO: motion onset, CP: choice point, LO: lick onset at goal well. **e**, Schematic of chance level calculation (see Methods). All five parameters are tested for the goal-well decoding, whereas only the direction and the random well selection were considered for the current-well decoding. **f**, Decoding performance of goal well using the following three kinematic variables together as predictors: acceleration (calculated as in Kropff et al^36^.), speed, and head direction (dotted line). The decoder was separately trained and tested on individual time points. The decoding performance based on the activity of OFC neurons is also included for comparison. Grey horizontal line denotes the times when the goal-decoding performance of the neuron-based decoder was significantly better than that based on kinematic variables (p < 0.05; two-sided Wilcoxon signed-rank test followed by Holm-Bonferroni correction, n = 18 sessions). **g**, Top: acceleration at motion onset at individual trials from a representative session plotted as a function of the distance to the goal (measured in a well-interval unit). The regression line best fitting the data is shown with the dotted line. The p-value of the regression slope is shown on top. Bottom: the regression slope between the acceleration at motion onset and the goal distance for all 18 sessions. Red asterisks denote sessions with statistically significant regression slope (p = 0.0015, 0.0065, 0.0885, 0.3239, 0.8152, 0.1810, 0.0289, 0.0024, 0.0744, 0.0087, 0.0008, 0.0025, 0.0042, 0.0717, 0.9600, 0.2889, 0.9802, 0.9101, from the t-statistic with the null hypothesis of zero slope without multiple comparison correction). **h**, Goal representation is largely independent of the animal’s speed or acceleration. Top left: for testing the effect of the animal’s speed at motion onset, we took an approach of grouping based on the animal’s running speed, whereby trials were divided into either two or four groups. Top right: we used the same strategy for testing the effect of the animal’s acceleration at motion onset. In both cases, we obtained almost the same chance levels between these two grouping strategies. Bottom left: goal decoding probability in trials with quick start using a decoder trained only on trials with slow start. Bottom right: data used in training and testing of a decoder were swapped. **i**, Decoding probabilities of ‘current’ well at different timepoints. Schematic on top depicts the time durations used for the decoder’s training (dark grey lines) with the class label of current well. Left: decoding of current well using the same strategy as used for the goal-well decoding (Figure 2d). Second left: identical to Figure 1h left but is included for comparison. Third and Fourth: decoding of current well relative to lick end and motion onset, respectively. The results together suggest that the current well is only weakly represented in OFC both at motion onset and during navigation, irrespective of the decoding strategy. MO: motion onset, LO: lick onset, LE: lick end. **j**, Decoding of current well as in Figure 1h, but with shorter durations of training data denoted in the schematic on top. **k**, Decoding probabilities and corresponding aggregate chance levels of the current (left 4 plots) or the goal (right 4 plots) well on a representative session from each rat. Same notations as in Figure 1h left or 2d left (trial number for the current or the goal well decoding: Rat 110 session 2, n = 137 and 119; Rat 175 session 5, n = 116 and 114; Rat 182 session 5, n = 149 and 146; Rat 284 session 5, n = 133 and 133). In panels **a–d**, **f**, and **h–k**, plots show means (line) ± s.e.m. (shaded).

**Extended Data Fig. 6:**
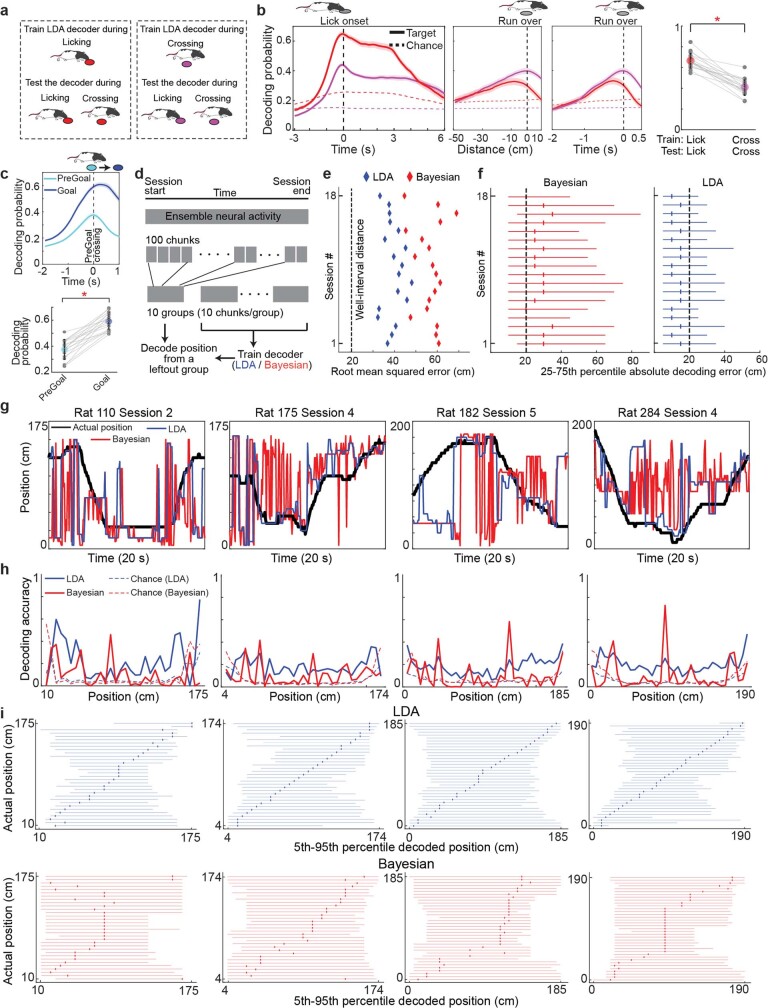
Decoding of the animal’s position during motion from ensemble activity of OFC neurons. **a–b**, Comparison of well representation of OFC neurons during licking versus crossing. **a**, Schematic of two different decoders. Left: a decoder was trained on the neural activity as animals approached and licked a target well (lick decoder). This decoder is the same as in Figure 1h. Right: another LDA-based decoder was trained on the neural activity as animals crossed a well without licking it (cross decoder). Training and testing for this cross decoder were performed in a 10-fold cross-validated manner, in which the entire session was divided into 10 equal-duration groups and the neural activity during well crossing from 9 groups were used to train a decoder, while the left-out group was used for testing. **b**, Left: decoding probability of licking well based on the two types of decoders trained during licking (red) or crossing (magenta). Middle two panels: distance (left) and time (right) based decoding of crossing well based on the two decoders. The red traces are identical to those in Figure 1h. Shown are means (line) ± s.e.m. (shaded). n = 18 sessions. Right: decoding probability of licking well using the decoder trained on the data during well licking, compared with that of crossing well based on the decoder trained on the data during well crossing. Results from individual sessions are shown in small grey circles while larger circles with error bars denote means ± s.d. (decoding probability of licking well: 0.67 ± 0.07; crossing well: 0.41 ± 0.08; *p = 1.96 × 10^−4^ in two-sided Wilcoxon signed-rank test), suggesting that well representation of OFC neural population is particularly strong during licking at goal wells. **c**, Top: decoding probabilities of goal well and its immediately preceding ‘pre-goal’ well when the animal crossed over the pre-goal well. The decoding of pre-goal well was performed using a decoder trained on cross-over wells (as in **a–b**), whereas goal-well decoding was performed by a decoder trained on a 2 s period prior to lick onset at goal wells. Shown are means (line) ± s.e.m. (shaded). Bottom: decoding probabilities of pre-goal and goal wells at the time of crossing the pre-goal well. Results from individual sessions are shown in small grey circles while larger circles with error bars denote the means ± s.d (decoding probability of pre-goal well: 0.37 ± 0.08; goal well: 0.59 ± 0.07; two-sided Wilcoxon signed-rank test: *p = 1.96 × 10^−4^). **d**, Schematic of the strategy to decode the animal’s instantaneous position from OFC neural population activity. As the animal perform multiple trial types with various start and goal positions during a session, the entire time duration of the session was first divided into 100 chunks of equal duration, and 10 groups were created by sampling 10 chunks per group randomly (without repetition), which ensure unbiased distributions of spatial bins among groups. To decode the spatial location, we then divided the animal’s position along the linear maze into 5 cm spatial bins. Spatial decoding was carried out on each group using 10-fold cross-validation, in which the neural activity during motion (speed > 10 cm/s) from 9 out of 10 groups was used to train a decoder while that of the left-out group was used for prediction of the rat’s location. Two types of decoding algorithms – LDA and Bayesian – were implemented. For LDA-based decoding, we trained a regularized LDA decoder (see Methods) with ensemble firing rate vectors at individual 100 ms bins using the class label of spatial bin occupied by the animal. For Bayesian decoding, we first calculated mean firing rates of each neuron at individual positions, and then estimated the posterior probability of the animal’s position at a particular spatial bin in a 100 ms bin using the following formula, $P({b}_{k})=C({\prod }_{i=1}^{N}({b}_{k}^{i}\,f){}^{{s}_{i}}){e}^{{-\sum }_{i=1}^{N}{b}_{k}^{i}f}$. where ${b}_{k}$ is the k^th^ spatial bin, C is a normalizing constant, N is the number of neurons, ${b}_{k}^{i}\,f$ is the average firing rate of the i^th^ neuron at the k^th^ spatial bin (calculated as the average number of spikes per 100 ms), and ${s}_{i}$ is the number of spikes fired by the i^th^ neuron during a given time bin. For both decoding strategies, the spatial bin with the highest probability was assigned as the decoded position. The decoders were trained on the activity of all neurons with mean firing rates greater than 0.5 Hz. **e**, Root mean squared decoding error for each session using the two decoding strategies (average root mean squared error for LDA and Bayesian: 39.51 ± 1.06 cm and 56.41 ± 1.49 cm respectively compared to the well spacing of 20 cm shown in a dotted vertical line; n = 18 sessions). **f**, Distribution of absolute decoding errors resulting from Bayesian (left) and LDA based (right) position decoding, shown as thin horizontal lines ranging from 25th to 75th percentile with ticks denoting the median. Each line represents data from one session. **g**, Decoded positions (vertical axis) during a 20 second period (horizontal axis) from four representative sessions. Positions decoded using LDA and Bayesian are shown in blue and red, respectively. **h**, Mean decoding accuracy, defined as a fraction of correctly decoded positions, for every spatial bin from representative sessions. Chance levels for LDA decoders were obtained by shuffling class labels during the decoder’s training. This procedure was repeated 100 times, generating a distribution of mean accuracies across spatial bins. Chance level for each bin was set at 95th percentile of this distribution. Similarly, for Bayesian decoding, chance levels were assessed based on shuffled firing rates among spatial bins. **i**, Distribution of decoded positions, shown as thin horizontal lines ranging from 5th to 95th percentile with ticks denoting the median, against the actual spatial location (vertical axis) occupied by the animal. Plots show the results of 4 representative sessions with a decoding strategy based on either LDA (top row) or Bayesian (bottom row).

Next, we asked how accurately OFC neurons represent the well position. We trained a decoder based on linear discriminant analysis (LDA) using the population activity of OFC neurons in the time range from 0.5 s before to 3 s after lick onset. The trained decoder was then applied to predict the well at which the animal was present (‘current’ well). The decoder predicted the current well above the chance from 1.7 s before to 6 s after lick onset (Fig. 1h, Extended Data Fig. 5). When the same decoder was applied to the wells that were passed through by the animal, it showed significantly lower performance (Fig. 1h, Extended Data Fig. 6). This result was also supported by poor performance of a decoder trained on the instantaneous position of the animal during running (Extended Data Fig. 6). We confirmed that the decoding of the current well was possible irrespective of the approach direction of the animal or the starting well (Fig. 1d, i), suggesting that OFC neurons form a largely viewpoint-invariant coding of spatial positions that are approached as navigational goals.

## Persistent goal representation in the OFC

**Fig. 2:**
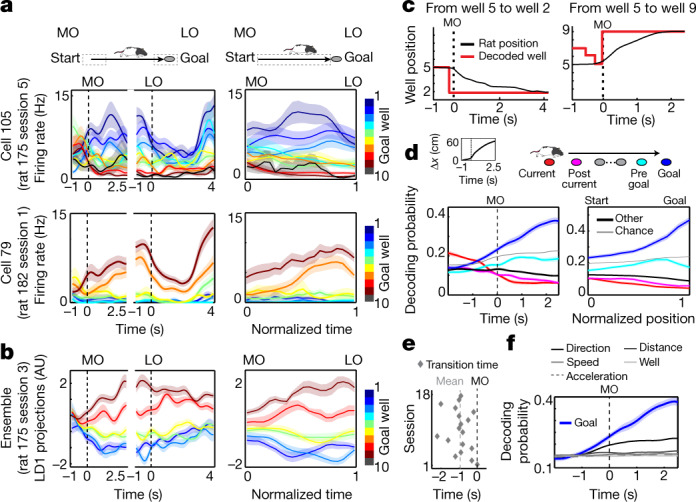
Persistent goal representation in the OFC. **a**, Firing of two representative neurons aligned to motion onset (MO) and lick onset (LO) or throughout navigation on normalized time (right). **b**, Same as **a**, except that the plots are based on ensemble neural activity projected on the axis with maximum goal well separability. **c**, Two representative trials showing the position of the animal (black) and the decoded well (red; well with maximum decoding probability). **d**, Decoding of goal well (blue) and current well (red), together with the wells next to current goal (magenta) or before goal (cyan), plotted over time from motion onset (left) or positional fraction of journey (right). The inset on the top left shows the displacement of the animal’s position. *n* = 18 sessions. **e**, Plot shows the times at which the decoding probability of the goal well first exceeded that of the current well. *n* = 18 sessions. **f**, Decoding of the goal well (as in **d**) compared to chance levels calculated on five null hypotheses (see Methods). In **a**, **b**, **d** and **f**, plots show mean (line) ± s.e.m. (shaded). AU, arbitrary units.

We then asked when the goal well representation develops in the OFC during navigation. We examined the firing rates of individual OFC neurons backwards in time towards the beginning of navigation and discovered that the rates at this time already differentiated the identity of the goal well (Fig. 2a). To confirm this observation at a neural population level, we projected the ensemble activity of OFC neurons on the dimension with maximal goal well separability calculated by LDA and found that the projected activity kept differentiating the next destination throughout navigation (Fig. 2b).

**Extended Data Fig. 7:**
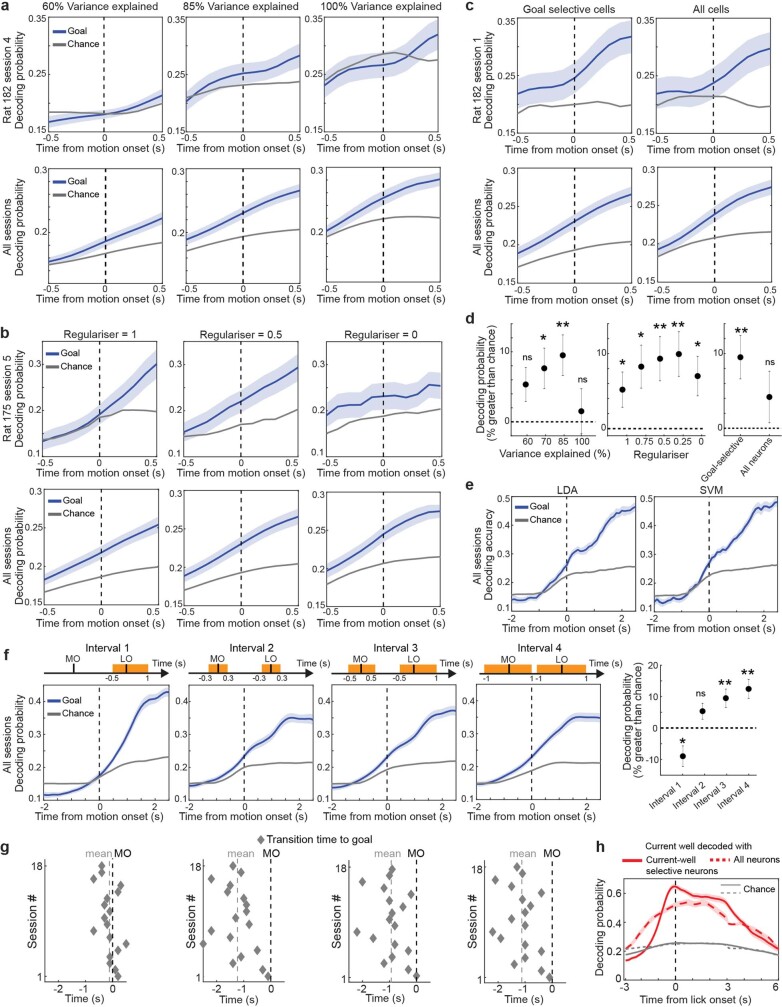
Choices of hyperparameters, decoding algorithm, and data range to optimize the goal decoding. **a**, Illustration of the impact of dimensionality reduction on goal-well decoding. This strategy, as well as the followings (**b** and **c**), were aimed to reduce the decoder’s dimensionality, as a decoder with a large number of parameters results in poor performance on a test dataset in general. Shown in each plot are the mean goal decoding probability and the corresponding chance level based on the data from a representative session (top) or across all sessions (bottom) (see Methods and Extended Data Fig. 5 for details). We implemented PCA to reduce the data dimensions to different degrees of explained data variance, and assessed the impact of dimensionality reduction on the performance of goal-well decoding. The decoding performance was optimal when the number of chosen dimensions explained 85% of the data variance, in terms of the maximum separation from the corresponding chance level as well as a small variance of the decoding probability. The decodings were performed with a fixed regularization value of 0.5 on goal-well selective neurons. **b**, Illustration of the impact of regularization. Shown are the decoding performances at three levels of regularization values. We found that the regularizer value of 0.5 has the maximum separation from the corresponding chance level as well as a low variance of the decoding probability. The decodings were performed with reduced dimensions explaining 85% of the data variance on goal-well selective neurons. **c**, Illustration of the impact of pre-selection of goal-well selective neurons. The decoding performance was assessed based on either goal-well selective neurons (left) or all recorded neurons (right). We found that the pre-selection of goal-selective cells achieved better separation from the corresponding chance level. The decoding was performed with the reduced dimensions explaining 85% of the data variance and the regularization value set to 0.5. **d**, Plots show a summary of decoding performance at motion onset relative to the corresponding chance level using different parameters described in **a–c**. *p < 0.05, **p < 0.01, and ns (p > 0.05). Left: p = 0.0936, 0.0156, 0.0084, and 0.9479; Middle: p = 0.0429, 0.0139, 0.0096, 0.0065, and 0.0279. Right: p = 0.0084, and 0.3061; in two-sided Wilcoxon signed-rank test without multiple comparison correction. n = 18 sessions. Errorbars denote s.e.m.). **e**, Comparison of decoding performance of goal well between two algorithms, LDA (left) and a support vector machine (SVM, right). For SVM we used a box-constraint of 0.01. Plotted are the decoding accuracy of goal well. For LDA, the predicted well was chosen as the one with the maximum probability. The two algorithms achieved similar decoding performance (the mean accuracy relative to the chance level at motion onset: LDA 0.0365; SVM 0.0432). **f**, Left: performance of decoders trained with different time ranges of neural activity. Four different ranges of the data were used as illustrated on top of each plot (orange bars). We found that the goal decoding improved by concatenating the neural activity at both motion onset and lick onset. Furthermore, the decoding performance at motion onset improved as a longer time range of the data was used for the decoder’s training. Interval 3 is the same as in Figure 2d. Right: summary of decoding performance at motion onset relative to the corresponding chance level for the four different decoding strategies described in left panel (n = 18 sessions, errorbars: s.e.m.). *p < 0.05, **p < 0.01, and ns (p > 0.05). p = 0.0176, 0.0642, 0.0084, and 0.0016 in two-sided Wilcoxon signed-rank test without multiple comparison correction. **g**, Plots showing the times when the decoding probability of the goal well exceeded that of the current well for each of the decoding strategies shown in panel (f) above. Grey diamonds indicate data from individual sessions (n = 18 sessions). Light vertical dotted line denotes the mean across sessions (interval 1: 0.12 ± 0.07 s before motion onset; interval 2: 1.24 ± 0.15 s before motion onset; interval 3: 0.93 ± 0.13 s before motion onset; interval 4: 1.12 ± 0.14 s before motion onset). **h**, Decoding probability of the well licked by the animal using only current-well-selective neurons (solid; see Methods for definition), compared with that from all neurons with the average firing rate greater than 0.5 Hz (dotted), demonstrating the improved decoding performance by the pre-selection of current-well selective neurons. In panels **a–c, e, f (left)**, and **h**, plots show means (line) ± s.e.m. (shaded).

Our findings of the OFC’s goal representation during navigation, as well as its coding of the position of the animal during reward consumption (Fig. 1h), imply a transition of spatial representation in the OFC before navigation onset. To identify such a transition, we took a decoding approach by training a decoder for goal well identity based on neural activity concatenated between the time segments at the beginning and the end of navigation (Methods). In individual trials, the decoder that initially indicated the start well of the trial exhibited an abrupt change in representation to the next goal well around the time of motion onset, which was then largely maintained during the entire journey (Fig. 2c). On the trial average, the activity of OFC neurons represented the well at which the animal was present (current well) until 0.7 s before motion onset (Fig. 2d, left). However, in contrast to the decay of the current well representation, the activity representing the goal well became significant from 1.1 s before motion onset (Fig. 2d, e, Extended Data Fig. 7), reaching a steady peak 2.5 s after the beginning of navigation (Fig. 2d, left, Extended Data Fig. 5). The decoding probability plotted along the positional fraction of navigation confirmed that the goal well was persistently represented throughout navigation (Fig. 2d, right).

**Extended Data Fig. 8:**
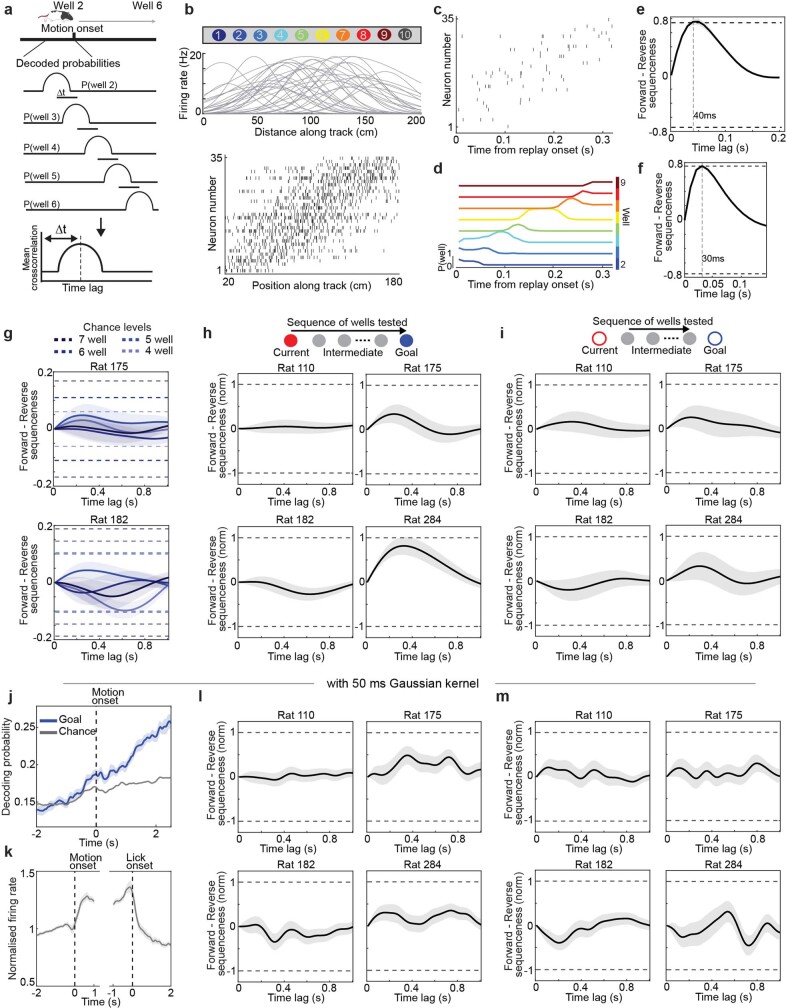
Non-sequential transition of spatial representations in OFC. **a**, Schematic of the technique to quantify the sequenceness of spatial representations. We here asked if OFC neurons exhibit sequential representations of spatial positions during a transition of their encoding position from the animal’s current location to its subsequent goal. We followed the technique described by Kurth-Nelson^37^, and examined whether the posterior decoding probabilities of wells obtained by the LDA decoder have sequential peaks. For example, when the spatial representation of OFC neurons switched from well 2 to well 6 prior to motion onset, we asked whether peaks of posterior probabilities of wells 3, 4, and 5 were observed in sequential order. We can test this possibility by examining the time lags of cross-correlations of decoding probabilities for individual wells. In the example case, we asked if we observed a consistent time lag for the peaks of cross-correlations between decoding probabilities of well pairs 2 and 3, 3 and 4, 4 and 5, or 5 and 6. We tested a possibility of both forward and reverse sequences (e.g., either from well 2 to well 6 or from well 6 to well 2, in the example). To account for autocorrelations, the difference between forward and reverse correlation is reported. Chance levels of sequenceness were calculated using a non-parametric method suggested by Kurth-Nelson et al^37^.. Briefly, the well identities were shuffled to obtain all possible combinations, for each of which the mean sequenceness was computed. For example, the sequence of wells in trials with the 4-well distance between the start and the goals can be shuffled in 120 different ways in total. Two of them represent the real forward and reverse sequences on the linear maze, and the other 118 are considered shuffled sequences. The maximum and minimum values from these shuffled sequences constitute the two chance levels (positive and negative) across time lags. **b–f**, Verification of the technique on simulated spike trains resembling hippocampal replay events. A virtual agent traversed a 2 meter long linear maze with 10 reward wells (well spacing of 20 cm) bidirectionally for 25 trials at a uniform speed of 25 cm/s. The agent travelled between the positions of 20 cm and 180 cm, thereby encountering wells 2 to 9 in every run. **b**, Top: gaussian spatial tuning curves of 35 simulated neurons. Position and peak firing rate were chosen from a uniform distribution ranging from 5 cm to 185 cm and 8 Hz to 20 Hz respectively. Bottom: spike raster plot of all simulated neurons in one of the simulated journeys from 20 to 180 cm along the maze. Spikes were generated in individual 100 ms bins assuming a Poisson process with the neuron’s position-dependent mean firing rates. **c**, Plot shows spike rasters during one replay event, out of 25 simulated events, in which each event comprised sequential representation of well locations from well 2 to well 9. We used a 20-fold time compression to simulate replay events^38^. Each well was represented for 40 ms, and firing rates of neurons were stretched over 10 cm from the centre of the well location. **d**, Posterior decoding probabilities of colour-coded individual wells (decoded using LDA, see Methods) from the representative replay event in **c**. Prior to decoding, spike trains were smoothed with 50 ms Gaussian kernel and binned at 10 ms. To classify a given well identity, the decoder was trained on the neural activity when the agent was within 5 cm of the corresponding well. **e**, Mean sequenceness across all simulated replay events (n = 25). As expected, the mean sequenceness exceeds the chance level at a time lag of 40 ms (dotted vertical line) corresponding to the average duration of individual well representations during the simulated replays. **f**, Mean sequenceness during a different simulation where the running speed of the agent was doubled, resulting in each well being represented for 20 ms during replays following a 20-fold compression. Our decoding strategy followed by the sequenceness detection algorithm was still able to detect this short-time sequential representation of positions, although inferring the precise timescale of well transitions appears to be prevented by the width of Gaussian kernel used for smoothing spike trains. **g**, Sequenceness algorithm applied to the posterior probabilities from −2 s to 0 s relative to motion onset from two representative animals. To identify the sequential transition of representation from the current to the goal well at a finer time scale, we binned neural activity into 10 ms time bins. We analysed trials where the start and the goal were separated by 4–7 wells. Plots show the difference between forward and reverse sequenceness for different trajectory lengths in distinct shades of blue along with their corresponding chance levels denoted by dotted lines. No significant sequenceness was observed in the representative animals. **h**, For clarity, the sequenceness for each trial was normalized so that the interval between the corresponding positive and negative chance level lied within 1 and −1, respectively. Using this normalization strategy, the sequenceness across trials with different distances of journeys could be pooled together. No overall significant forward or reverse sequenceness was observed in any of the four animals used. **i**, Same as in **h** except that the sequenceness was calculated with the middle wells of journey without the current and goal wells in order to exclude possible artefacts due to overrepresentations of these wells. For this analysis, we only focused on trials where starts and goals were separated by 6-7 wells. **j–m**, As sequential transitions in neural states may occur at a finer time scale in the order of a few tens of ms, we reanalysed our neural data by convolving spike trains with a 50 ms Gaussian kernel (rather than 250 ms), which matches the condition of our simulations in **b–f**. **j**, Decoding probability of the goal well relative to motion onset. Goal wells can be decoded greater than chance levels from 1.4 s prior to motion onset. **k**, Plot shows instantaneous firing rates of goal-well selective OFC cells. Firing rates were normalized to the means over the session. Unlike place cells that exhibit elevated instantaneous firing rates during replay events, we did not find any increase in instantaneous firing rates prior to motion onset. **l–m**, the same plots as in **h–i** except for the use of the 50 ms Gaussian kernel. No significant forward or reverse sequenceness was observed. In panels **e–m**, plots show means (line) ± s.e.m. (shaded).

To confirm the decoder’s selective representation of goal well over others, we assessed the representations of wells that were passed through by the animal during navigation, particularly the wells that immediately followed the start or preceded the goal along the journey. We found that the decoding probabilities of these wells with the goal well decoder were significantly lower than that of the goal well throughout the course of navigation (Fig. 2d). Additional analyses further confirmed that goal decoding was not due to other task-associated factors (Fig. 2f) and that the transition of representation from the current well to the goal well did not involve sequential activation of neighbouring positions^3,10,11^ (Extended Data Fig. 8). These results together suggest that the activity of OFC neurons switches their spatial representation from the starting position of the animal to the next destination before navigation onset, subsequently maintaining it throughout navigation.

We further investigated the goal representation in error trials. We applied the goal well decoder trained on correct trials to neural activity at the beginning of error trials and found that it could decode the subsequent incorrect destination of the animal as accurately as goal wells in correct trials (Extended Data Fig. 5). The activity of OFC neurons thus represents the next target well of the animal irrespective of its correctness, reflecting the animal’s decision of goal destination.

## Goal coding is orthogonal to OFC dynamics

**Fig. 3:**
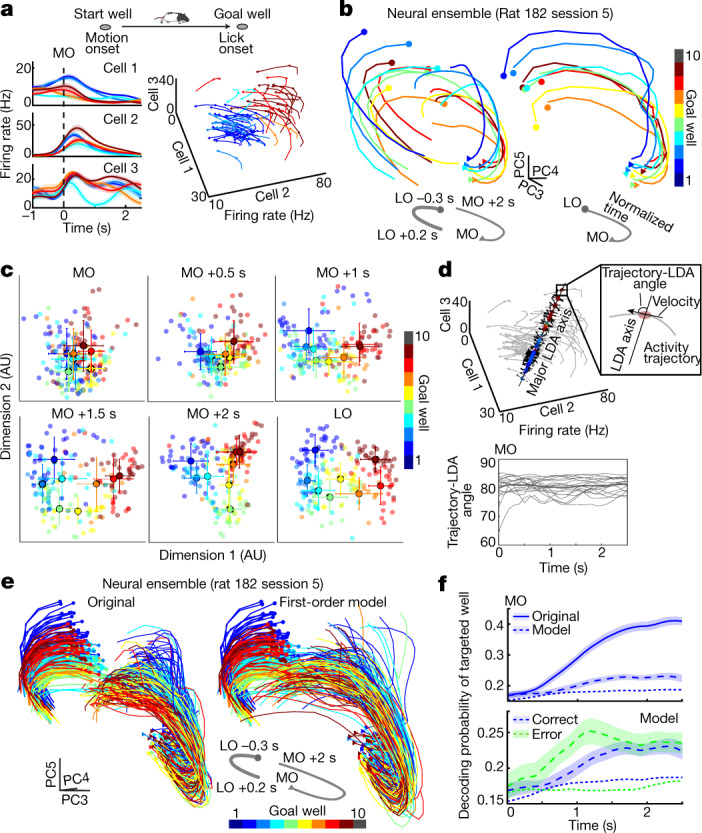
Orthogonal coding of spatial goals to evolving OFC dynamics. **a**, Illustration of dynamic coding. Left, firing of three neurons aligned to motion onset. Right, firing of the same neurons plotted on individual axes of a three-dimensional space, forming similarly shaped activity trajectories separated from each other depending on future goals. **b**, Ensemble activity of OFC neurons in reduced dimensions using PCA plotted separately aligned to motion and subsequent lick onsets (left) or in normalized time (right). Shown are trial averages based on goal wells. **c**, Ensemble neural activity in individual trials projected on the axes with maximal goal well separability using LDA, calculated at individual time points and reduced to two dimensions using Isomap^30^. Opaque circles with error bars denote mean ± s.e.m for each well. **d**, Relationship between the evolution of dynamics and the goal well separability. Top, as in **a**, along with the axis of maximum well separability (first LDA dimension). Instantaneous velocities of neural trajectories are shown with arrows. Bottom, plot shows angular differences (in degrees) between the velocity vectors and the major LDA axis from individual 18 sessions (thin) and means (thick). **e**, Plot shows three principal components (PCs) of neural activity trajectories from individual trials extracted using LDA (Methods). Left, original trajectories from neural data, separately aligned to motion onsets (thin) and lick onsets (thick). Right, simulated trajectories from the first-order linear dynamic model fed with neural activity at motion onset. **f**, Top, goal decoding from the real (original) and the simulated (model) trajectories. Bottom, destination decoding between correct and error trials from the simulated trajectories. Dashed lines indicate chance levels. In **a** and **f**, plots show mean (line) ± s.e.m (shaded). *n* = 18 sessions. AU, arbitrary units.

**Extended Data Fig. 9:**
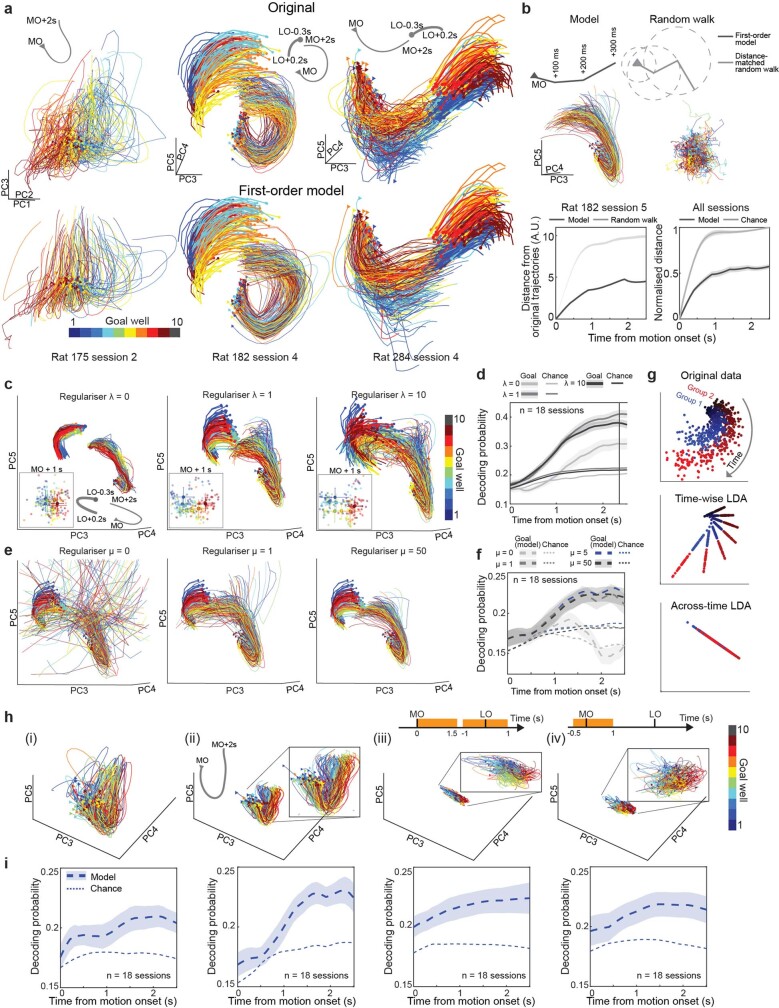
Choice of hyperparameters and LDA-based denoising strategy for analysing destination-specific neural dynamics. **a**, Plots showing neural activity trajectories from individual trials extracted using LDA (as in Figure 3e) from three representative sessions. The trajectories are colour-coded based on the animal’s destination. Top: original trajectories from the neural data, separately aligned to motion onset (MO; thin) and subsequent lick onset (LO; thick). Bottom: simulated trajectories with a first-order linear dynamic model based on the neural activity at motion onset. Trajectories aligned to lick onset were omitted from the left panel (Rat 175) to facilitate visualization. **b**, Quantification of the accuracy of first-order linear models. Top: schematic of a random walk model. At each time step, a first-order model predicts a displacement vector based on the activity state at a given time. Iterative additions of these displacement vectors to the neural activity at motion onset result in a predicted neural activity trajectory. We hypothesized that a fair null model to test our first-order model would be a distance-matched random-walk model, in which each displacement vector obtained from the first-order model is randomly rotated, thereby preserving the magnitude of displacement at each time step. Middle: example trajectories generated by the first-order model (left) and its distance-matched random-walk model (right) using data from the same representative session as in Figure 3e. Bottom left: average Euclidean distance between the modelled and the original trajectories (dark, n = 146 trials) from the representative session. The light shaded region denotes the full distribution of distances between the original and the simulated trajectories by 1000 random-walk models. Bottom right: normalized average Euclidean distance between the original and the modelled trajectories across all 18 sessions. For each session, all the distances (both the modelled and random trajectories) were normalized to the minimum distance generated by the random-walk models at the time point of 2.5 s after motion onset. Chance level at a given time point for each session was set at the smallest normalised distances between the original and random-walk-model-generated trajectories. **c**, Effect of regularization on LDA-based projections of neural activity from a representative session (same as in Fig. 3). The plots show three principal components (PC) of activity trajectories from individual trials extracted using LDA with different regularization values (see Methods). The trajectories are colour-coded based on the animal’s destination and are separately aligned to motion onset (thin) and lick onset (thick). Insets show the ensemble neural activity in individual trials during one second after the motion onset projected on the axes that maximize the goal separability. The ranges of PC axes are the same across the panels. Absence of regularization caused overfitting, resulting in poor generalization and goal separability. In contrast, a large regularization value (e.g., $\lambda$ = 10) separated data primarily based on class means with minimal influence of within-class covariance, resulting in suboptimal separation. We thus chose an intermediate regularization value of 1. **d**, Probability of goal-well decoding from neural trajectories extracted using LDA with three different regularization values. The decoding strategy was the same as in Figure 3f (also see Methods). Decoding performance, assessed as the difference between the mean goal decoding probability and the corresponding chance level, was optimal at λ = 1, which was used in the rest of the analyses. Shown are means (solid) ± s.e.m. (shaded). n = 18 sessions. **e**, Neural trajectories from a representative session simulated with a first-order linear dynamical model using three different regularization values. The ranges of axes are the same across the panels. Without regularization, simulated trajectories expanded quickly beyond the range of original neural activity, whereas a high regularization value constrained the models to simulate relatively simple trajectories. We found that regularizer values between 1 and 5 obtained the models that simulate activity trajectories similar to the original data. **f**, Probability of goal decoding from neural trajectories simulated with different regularization values. Optimal decoding performance was obtained with a regularization value of either 1 or 5, and we thus chose µ = 5 for the rest of the analyses. For the regularization of µ = 5, s.e.m. is not shown in the plot for better presentation of other results, but it is shown in panel **i**(ii) and Figure 3f. **g**, Demonstration of the advantages of performing LDA at individual time points based on simulated data. Top: temporal evolution of two groups (red and blue) of Gaussian distributed data evolving with first-order linear dynamics. Data in progressive time steps are coloured with incrementally lighter shades. Middle: data points projected to multiple LDA axes calculated at different time steps, which preserves the dynamics while keeping the separation between the two groups. Bottom: data projected to a single LDA axis calculated from the data across trial durations, which failed to preserve both the dynamics and the optimal group separation. **h**, Ensemble neural activity from a representative session extracted using different LDA-based denoising strategies (see Methods): (i) Original neural activity, (ii) Neural activity extracted using multiple LDA subspaces evaluated at individual time points, (iii) and (iv) Neural activity extracted by a single LDA subspace evaluated by concatenating two different time ranges of the neural activity (orange lines). Only neural trajectories aligned to motion onset (MO) are shown. The ranges of axes are the same across the plots. Insets provide magnified views of compact neural activities. Although the activity extraction with a single LDA subspace failed to preserve the original neural dynamics (iii and iv), implementation of multiple LDAs at individual time points succeeded in extracting destination-specific trajectories by preserving the original dynamics (ii). **i**, Probability of goal-well decoding based on the neural trajectories extracted by individual strategies corresponding to i-iv in **h**, demonstrating the optimal decoding performance of the time-wise LDA-based extraction method (ii in **h**).

Although our decoding analysis indicates a persistent goal representation in the OFC, firing rates of individual neurons changed markedly during navigation (Fig. 1b), implying that the encoding of goal locations in the OFC is not through the convergence of neural activity towards a point attractor but likely by dynamic coding evolving over navigation (Fig. 3a). We implemented a principal component analysis (PCA) to obtain reduced dimensions of neural population activity in the OFC. We found that activity trajectories, averaged over trials based on subsequent goals, exhibited similar dynamics while maintaining separation between each other (Fig. 3b). To understand how the goals of individual trials are embedded in activity trajectories, we applied an LDA-based dimensionality reduction approach to obtain the best projections of population activity for goal well selectivity at different times of navigation (Methods, Extended Data Fig. 9). The goal well separation was largely maintained during navigation, albeit it transformed from a compact to a distributed configuration as the animal approached the destinations (Fig. 3c). We also found that the major axis of goal well separation was nearly orthogonal to the direction of activity trajectories (Fig. 3d), suggesting that goal locations are encoded largely independently of the evolution of dynamics. We, therefore, asked whether the dynamics of individual trials could be modelled independently of their goal selectivity. First, the destination-specific activity extracted by LDA was projected back to the original neuronal dimensions, forming time courses of goal-dependent dynamics by minimising activity irrelevant to goal coding (Fig. 3e, left). We then fitted a first-order linear dynamic model on neural activity trajectories of a 2.5-s duration from motion onset to capture the global trend of dynamics irrespective of destinations (Methods). Finally, the constructed model was fed with the neural activity at motion onset, generating simulated trajectories up to 2.5 s afterwards. We found that the simulated trajectories evolved in a similar manner as the original ones (Fig. 3e, right), which was confirmed by the improvement of goal well decoding over the time course of navigation (Fig. 3f). We further found that our first-order model, trained on correct trials, was also able to simulate the neural activities on error trials, in which the activity evolved to indicate the incorrectly visited destination of the animal (Fig. 3f). OFC neurons thus encode the destination of the animal orthogonally to the ensemble dynamics that evolve, at least in part, deterministically from navigation onset.

## OFC perturbation led to a navigational error

**Fig. 4:**
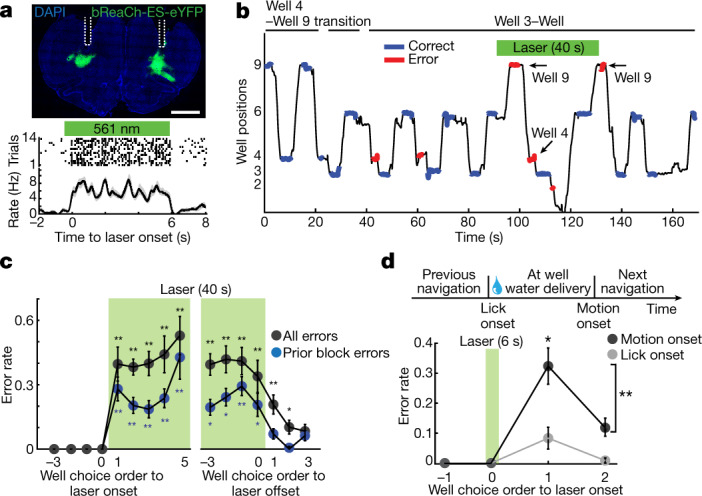
OFC perturbation impairs accurate navigation. **a**, Top, coronal section showing expression of bReaCh-ES-eYFP (green). Dotted white lines indicate the positions of optic fibres. Bottom, plot shows spike rasters and mean rates of a representative OFC neuron during a 6-s laser pulse train (15-ms pulses at 5 Hz). *n* = 14 trials. Scale bar, 2 mm. **b**, Plot shows a representative behaviour of the bReaCh-ES-expressing animal. The position of the animal is plotted over time (black line), together with the licking of the animal at either correct (blue) or incorrect (red) wells. Previous block errors are shown with arrows. **c**, Error rates of the animals before, during and after a 40-s laser pulse train (mean ± s.e.m.). Errors to the wells rewarded in the previous block are shown separately. **P < 0.01 or *P < 0.05 in two-sided Wilcoxon signed-rank test with post hoc Bonferroni correction. *n* = 12 sessions from three rats. **d**, Error rates of the animals subjected to a 6-s laser pulse train applied at motion onset (black) or lick onset (grey). Error bars denote s.e.m. *n* = 9 sessions from three rats. *P = 0.020 or **P = 0.008 in two-sided Wilcoxon signed-rank test.

**Extended Data Fig. 10:**
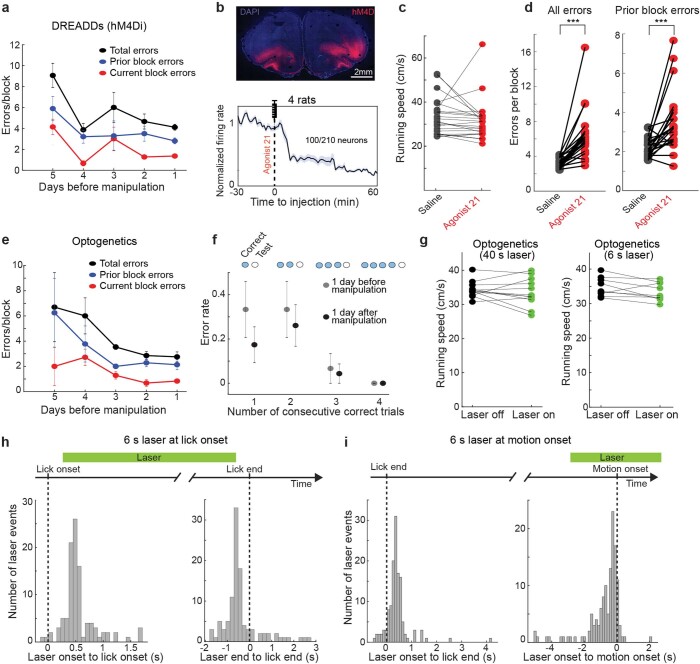
DREADDs-mediated manipulation of OFC neurons and additional analyses for optogenetic perturbation experiments. **a**, Mean number of errors per block committed by the animals injected with AAV8-hSyn-hM4Di-mCherry (left, n = 4 rats; a gift from Bryan Roth; Addgene viral prep # 44362-AAV8) five days prior to the beginning of perturbation experiments. The volume of 500 nL was injected at eleven sites in the OFC of each hemisphere with the following coordinates (AP, ML, and DV in mm): 2.7, 3.5, 5.2; 3, 2.5, 4.6; 3, 3.8, 4.4; 3.6, 2, 4.2; 3.6, 3.6, 4; 4.2, 1.4, 4.2; 4.2, 2.4, 4; 4.7, 1.6, 3; 4.7, 2.8, 3.5; 5.2, 1.2, 2.6; and 5.2, 2.6, 2.6. To evaluate the effects of manipulation, a microdrive with two circular bundles of 6 movable tetrodes each was implanted bilaterally with the centres of the bundles positioned at 3.5 mm (AP) from bregma and 1.5 mm (ML) from midline. Total errors and the two major error types — prior block errors and current block errors (defined in Extended Data Fig. 1)— are plotted. Shown are means ± s.e.m. **b**, Top: coronal section showing expression of hM4Di-mCherry in bilateral OFC. Bottom: normalized firing rates of OFC neurons over time relative to the subcutaneous injection of Agonist 21 (DREADDs Agonist 21 dihydrochloride, 7.04 mg/mL [20 mM]; Hello Bio at a dose of 6 mg/Kg). Means (solid) ± s.e.m. (shaded) across 100 neurons. **c**, Average speed of the animals expressing hM4Di-mCherry during motion (speed > 10 cm/s) when injected with saline versus Agonist 21. Black oblique lines represent paired sessions (see Methods). **d**, Plot shows the number of errors per trial block in the saline (grey) or Agonist 21 (red) injected sessions. The animals were injected with Agonist 21 followed by at least 45 min waiting time to allow the drug to reach the brain and take effect before starting the behaviour sessions. On control days, the equal volume of 0.9% saline solution was injected. To evaluate the impact of OFC silencing, the same sequences of well combinations were tested in a pair of saline and Agonist 21 sessions. The two sessions were carried out on consecutive days in a randomized order. All types of errors (left; ***p = 3.08 × 10^−5^ in two-sided Wilcoxon signed-rank test: z = −4.16) and the errors to the wells rewarded in the previous block (right; *** p = 3 × 10^−4^ in two-sided Wilcoxon signed-rank test: z = −3.61,) are shown separately. n = 23 sessions from 4 animals injected with AAV encoding hM4Di-mCherry. **e**, Mean number of errors per block committed by the animals injected with AAV1-CamKII-bReaCh-ES-eYFP five days prior to the start of perturbation experiments. Shown are means ± s.e.m. (n = 3 rats). **f**, Average error rates following consecutive correct licks in a block one day before and after the optogenetic perturbation experiments. The horizontal axis indicates the number of consecutive correct trials prior to the trial being evaluated. All the three animals made no errors after 4 consecutive correct trials, and thus we performed optogenetic perturbations after the first four consecutive correct trials in a block. Furthermore, after the termination of perturbation, the animals still did not make any errors after four consecutive correct trials, suggesting that this criterion is most likely valid during the entire course of perturbation experiments. Shown are means ± s.e.m. (n = 3 rats). **g**, Average running speed of the animals expressing bReaCh-ES-eYFP during the laser pulses of 40-s duration (left; running speed: laser on 33.54 ± 1.18 cm/s, laser off 34.37 ± 0.68 cm/s; p = 0.38 in two-sided Wilcoxon signed-rank test; n = 12 sessions; analyses were restricted during motion [speed > 10 cm/s]) or 6-s duration (right; running speed: laser on 33.4 ± 0.86 cm/s, laser off 34.85 ± 0.92 cm/s; p = 0.074 in two-sided Wilcoxon signed-rank test; n = 9 sessions). Each point in the plots represents the average speed from one session. **h**, Histogram of the times of either laser onsets relative to lick onset (left) or laser ends relative to lick end (right) in the experiments with 6 s optogenetic perturbation at lick onset. The vertical axis indicates the number of laser events, and the horizontal axis represents time relative to lick onset (left) and lick end (right). 98% (102 out of 104) of laser onsets occurred after lick onset and 83.65% (87 out of 104) of laser pulses ended before lick end. **i**, Histogram of the times of laser onsets relative to either lick end (left) or motion onset (right) in the experiments with 6 s optogenetic perturbation at motion onset. 91.79% (123 out of 134) of laser onsets occurred after lick end, and 95.52% (128 out of 134) of laser pulses started within 100 ms relative to motion onset. Three laser events that started 5 s after lick end, as well as six laser events that started more than 6 s prior to motion onset, were excluded from the plots.

Finally, we asked whether the activity of OFC neurons causally influences the destination of the animal. We first confirmed that pharmacogenetic inactivation of OFC neurons resulted in a significant increase in the animal’s incorrect choices of destination (Extended Data Fig. 10). To address whether the effect of perturbation is specific to ongoing navigation, we injected adeno-associated virus (AAV) encoding the excitatory opsin bReaCh-ES-eYFP^12^ followed by implantation of optic fibres in the bilateral OFC in three rats (Fig. 4a). We chose the frequency and power of stimulation that elicited reliable spiking in OFC neurons without affecting the motion of the animal (Extended Data Fig. 10). When laser pulses were applied for a 40-s duration, the animals started making more errors immediately after the onset of perturbation (Fig. 4b, c), which gradually subsided after the termination of laser pulses.

We then asked whether the effect of perturbation is stronger during the development of goal representation in the OFC. To explore this possibility, we applied laser pulses of 6-s duration either at motion onset or at lick onset (Extended Data Fig. 10) and found that the pulses applied at motion onset caused more errors in the subsequent navigation of the animal (Fig. 4d). This deficit was largely recovered in the immediately succeeding trial, suggesting that the impairment is not due to general loss of goal memory. The activity of the OFC is thus crucial for determining the next destination of the animal, particularly at navigation onset when a goal representation develops in its ensemble activity.

## Discussion

In this study, we identified the OFC as a brain region that represents the subsequent destination of an animal throughout navigation. Neural activity correlated with the goal-directed trajectories of animals has been previously described in the rodent hippocampus in the form of brief sequential firing among place cells^5,6^. However, this activity encodes not only a particular location of interest but also its nearby positions due to the sequential nature of hippocampal spatial coding^13^, and several studies have cast doubt on its role in determining the destination of animals^8,9^. Studies in human subjects, by contrast, described the activity modulation in the hippocampus that depends on the next destination instructed by a given cue^14,15^. It is yet unclear whether this modulation represents the goal location itself or its associated instructive cue or how it can be integrated with a hippocampal spatial map to point to the exact goal position. Goal coding in the OFC is different. The activity of OFC neurons encodes accurate well positions on the maze and exhibits an abrupt transition of encoding position from the location of the animal to its destination without relying on external sensory cues. This goal representation, developed before navigation onset, is then maintained throughout navigation without representing nearby positions or trajectories.

The prefrontal cortex has been considered a key area for navigation^16^. Lesioning of its ventromedial region, including the OFC, has been reported to impair accurate targeting to a destination in humans and rats^17,18^. The activity of the OFC is modulated during goal-directed motion^19,20^ or navigational planning^15^, and we discovered, in this study, that it can form a representation of spatial goals. The decision of navigational goal requires a choice among available positions. This is consistent with a previously suggested role for the OFC in choice decisions based on prior history of choices and subsequent outcomes^21–24^. The representation of spatial goal, however, needs to satisfy additional cognitive demand for navigation. Although accurate coding of spatial position requires sensory and proprioceptive inputs, the emergence of goal representation, as well as its persistence during navigation, indicate suppression of these inputs along the goal-directed journey. This resistance of goal representation to incoming inputs appears to be achieved by dynamic coding. Unlike place cells^1^ or grid cells^4^, OFC neurons discriminate positions in a dynamic manner, whereby neural activity changes during navigation while optimising the separation of encoded destinations. Dynamic coding of behavioural variables has been described in many brain regions and species^25–28^ and is thought to minimize interference between orthogonal neural activity subspaces^28,29^. Goal coding in the OFC might then be used in downstream circuits to form goal-directed trajectories, enabling animals to navigate from one location to another by relying on a cognitive map.

## Methods

### Subjects

All experiments were approved by the local authorities (RP Darmstadt, protocols F126/1009 and F126/1026) in accordance with the European Convention for the Protection of Vertebrate Animals used for Experimental and Other Scientific Purposes. Nineteen male Long-Evans rats weighing 400–550 g (aged 3–6 months) at the start of the experiment were housed individually in Plexiglass cages (45 × 35 × 40 cm; Tecniplast, GR1800) and maintained on a 12-h light/dark cycle, with behavioural experiments performed during the dark phase. For experiments in the linear maze, the animals were water restricted with unlimited access to food and kept at 90% of their free-feeding body weight throughout the experiment. For recordings in the open-field arena, the animals were food restricted with unlimited access to water and kept at 85–90% of their free-feeding body weight. Four of the rats had tetrodes implanted in the OFC, and one had tetrodes implanted in the hippocampus. Two rats had a silicon probe (Buzsaki64sp, NeuroNexus) implanted in the OFC, which was used for recordings in a modified T-maze task (Extended Data Fig. 5d). Seven rats received AAV injections in the OFC—four of them for designer receptors exclusively activated by designer drugs (DREADD) experiments and three for optogenetic experiments. Five rats, used only for behaviour analysis, received a metal implant on their skull to hold LEDs for position tracking. No statistical method was used to predetermine the sample size.

### Surgery, virus injection and drive implantation

Anaesthesia was induced by isoflurane (5% induction concentration, 0.5–2% maintenance adjusted according to physiological monitoring). For analgesia, Buprenovet (buprenorphine, 0.06 mg ml^−1^; WdT) was administered by subcutaneous injection, followed by local intracutaneous application of either bupivacain (bupivacain hydrochloride, 0.5 mg ml^−1^, Jenapharm) or ropivacain (ropivacain hydrochloride, 2 mg ml^−1^, Fresenius Kabi) into the scalp. Rats were subsequently placed in a Kopf stereotaxic frame, and an incision was made in the scalp to expose the skull. After horizontal alignment, several holes were drilled into the skull to place anchor screws, and craniotomies were made for microdrive implantation. The microdrive was fixed to the anchor screws with dental cement, and two screws above the cerebellum were connected to the electrode’s ground. All animals received analgesics (Metacam, 2 mg ml^−1^ meloxicam, Boehringer Ingelheim) and antibiotics (Baytril, 25 mg ml^−1^ enrofloxacin, Bayer) for at least 5 d after the surgery.

For tetrode recordings, rats were unilaterally implanted with a microdrive that contained individually adjustable tetrodes made from 17-mm polyimide-coated platinum–iridium (90–10%, California Fine Wire, plated with gold to impedances below 150 kΩ at 1 kHz). The tetrode bundle consisted of 30-gauge stainless steel cannulae, soldered together in circular or rectangular shapes. The drives were implanted in the OFC of the left hemisphere in four rats with the following coordinates and bundle designs: Rat 110 with a 14-tetrode rectangular bundle (anterior–posterior (AP): 2.75–4.5 mm, medial–lateral (ML): 1.5–2.5 mm alongside the anteroposterior axis); Rat 175 with a 28-tetrode rectangular bundle (AP: 2.75–4.9 mm, ML: 1.2–2.7 mm); Rat 182 with a 42-tetrode rectangular bundle (AP: 2.75–5 mm, ML: 1.2–3.0 mm); and Rat 284 with a 42-tetrode circular bundle (AP: 2.75–5.25 mm, ML: 1.0–3.5 mm). Tetrodes were implanted at an initial depth of 2 mm dorsoventral (DV) from the dura and progressively lowered to the final depths of 2.5–4.5 mm. For the recording from neurons in area CA1 of the hippocampus (Extended Data Fig. 4), a circular bundle of 14 independently movable tetrodes was implanted in the right hemisphere (AP: −3.5 mm, ML: 3.5 mm). For the recording from neurons in the OFC in a modified T-maze task (Extended Data Fig. 5d), a silicon probe was implanted in the right hemisphere (AP: 3.5 mm, ML: 2 mm). Experiments began at least 1 week after the surgery to allow the animals to recover.

For optogenetic perturbation of OFC neurons, AAV1-CamKII-bReaCh-ES-EYFP (a gift from K. Deisseroth)^12^ was injected into three sites in both hemispheres of the OFC (AP, ML and DV in mm: 3, 3, 4.5; 3.5, 2.8, 4.25; 4, 2.5, 4, respectively). The AAV was injected with an infusion rate of 100 nl min^−1^ using a 10-ml NanoFil syringe and a 33-gauge bevelled metal needle (World Precision Instruments). After injection was completed, the needle was left in place for 10 min. The volume of 500 nl was injected at each site. Two optic fibres (FP400URT, Thorlabs) were implanted with their tips positioned at approximately 500 µm above the OFC of both hemispheres (AP: 3.5 mm, ML: 2.8 mm and DV: 3.25 mm). The optic fibre in the left hemispheres had two tetrodes attached, with their positions advanced approximately 750 µm from the fibre tip to monitor the neural activity nearby. The virus injection and the optic fibre implantation were performed in the same surgery, and experiments started at least 4 weeks after the surgery.

### Behavioural methods

Rats were trained in the 2-m-long linear maze with ten reward wells distributed at an equal distance (20 cm) between each other. The training procedure consisted of three phases. In the first phase, 100 µl of liquid reward (0.3% saccharin) was manually delivered at two specific wells alternately. Most rats learned to lick wells within 2 d of training. In the second phase, rewards were delivered only after the rat licked the correct wells, but, here, a reward was delivered immediately after the animal’s correct lick. The training duration for this phase lasted for 1–7 d, depending on the individual rats. In the final phase, a transition rule was introduced. Once the rat made at least six consecutive correct trials, rewards were delivered in a new pair of wells, which was signalled by LEDs, positioned directly underneath all the ten wells on the maze, together with the delivery of water rewards at the new well pair. These LEDs thus did not give any position information, and the new goal wells were pre-filled with water before the animal’s approach. The LEDs turned off once the animal consumed these rewards. Furthermore, the animal was required to keep licking the correct well for a fixed amount of time, defined as lick threshold, for a reward to be delivered. Of all the 18 neural recording sessions, the lick threshold was set to 2 s for 12 sessions, 1.5 s for one session and 1 s for five sessions, respectively (Extended Data Fig. 1). The lick threshold was set to 1 s for all DREADD-mediated silencing, optogenetic perturbation and modified T-maze experiments. The licking of the animal was continuously monitored by infrared sensors (Turck) equipped on individual wells, and, once the duration of the animal’s licking exceeded a pre-defined threshold, a tone was generated, followed immediately by the delivery of water with a peristaltic pump (Cole-Parmer). The details of licking behaviours are shown in Extended Data Fig. 1f–h, and the difference of lick threshold did not affect the decoding performance significantly (Extended Data Fig. 1i).

The behavioural analyses (Extended Data Fig. 1) started from the first day of phase 3 training, and each session lasted for 30 min. Neural recording sessions were carried out after the animals reached steady levels of behavioural performance (with stable prior block error rates over a period of three consecutive days—usually achieved within 15 d of training). Trials during the transition to a new well pair were discarded from the analyses. Although one of the rewarded wells in one block could be rewarded again in the immediately succeeding block, this did not affect the learning rate of the animal compared to the blocks where both goal wells were changed (Extended Data Fig. 1j). The number of wells used in each recording session was as follows (out of all ten wells): ten wells in one session, eight wells in five sessions, seven wells in eight sessions and six wells in four sessions. The position and head direction of the animal were monitored with two-coloured LEDs on the head stage at the sampling rate of 25 Hz. All the recordings were performed under a minimum-light condition (no light source in the recording room, with only weak ambient light coming from the adjacent room from computer monitors).

For optogenetic experiments, laser pulses (15-ms width at 6 Hz) were generated from a 561-nm DPSS laser unit (Dragon Laser) for a fixed amount of duration of either 40 s or 6 s. The laser power at the fibre tip in each hemisphere was 1.5 mW. The onset of laser pulses was manually triggered based on the behaviour of the animal on the task, and the time stamps of the pulses were recorded. Perturbation experiments were performed after the animals reached steady levels of behavioural performance (observed as stable prior-block error rates over 3 d; Extended Data Fig. 10).

### Histological procedures

Once the experiments were completed, the animals were deeply anaesthetized by sodium pentobarbital and perfused intracardially with saline, followed by 10% formalin solution. The brains were extracted and fixed in formalin for at least 72 h at 4 ºC. Frozen coronal sections were cut (30 µm) and stained using cresyl violet and mounted on glass slides.

### Spike sorting and cell classification

All data processes and analyses were performed with MATLAB (MathWorks). Neural signals were acquired and amplified using two or four 64-channel RHD2164 headstages (Intan Technologies), combined with an OpenEphys acquisition system with the sampling rate at 15 kHz. The signals were band-pass filtered at 0.6–6 kHz, and spikes were detected and assigned to separate clusters using Kilosort^31^ (https://github.com/cortex-lab/KiloSort) under the parameter settings of the spike threshold at −4 and the number of filters at 2× the total channel number. Each tetrode was independently grouped with ‘kcoords’ parameters, and the noise parameter determining the fraction of noise templates spanning across all channel groups was set to 0.01. The obtained clusters were checked and adjusted manually based on autocorrelograms and waveform characteristics in principal component space, obtaining well-isolated single units by discarding multi-unit activity or noises. Neurons with firing rates less than 0.5 Hz were excluded. Spike times were converted into firing rates, except for the analyses for the open-field experiment (Extended Data Fig. 4) and the conjunctive coding of spatial location and navigation phase (Fig. 1e, Extended Data Fig. 3). The firing rate estimation was performed by convolving spike times by a Gaussian kernel with a bandwidth of 250 ms.

### Cell classification

#### Spatial selectivity

Firing rates of a neuron were assessed at individual spatial bins of 10 cm along the linear maze across trials. For each spatial bin, random sampling was performed 100 times at various epochs of the session, either when the animal was moving (running speed >10 cm s^−1^) or not moving (running speed <10 cm s^−1^), obtaining 200 samples of firing rates per spatial bin (Extended Data Fig. 3). By concatenating these samples across the bins, we created the firing rate distributions of 200 pseudotrials along the maze and evaluated the consistency of spatial tuning by computing pairwise dot products between them. The average of the dot products was considered as a representative value of spatial tuning of the cell. For the corresponding null hypothesis, we shuffled the neural activity between spatial bins for individual pseudotrials and calculated the average dot product between them. This entire process of generation of pseudotrials, as well as calculation of the average dot products for the real and shuffled data, was repeated 1,000 times. The difference between the two distributions was quantified as follows:

$$z=\frac{{\mu }_{{\rm{r}}{\rm{e}}{\rm{a}}{\rm{l}}}-{\mu }_{{\rm{s}}{\rm{h}}{\rm{u}}{\rm{f}}{\rm{f}}{\rm{l}}{\rm{e}}{\rm{d}}}}{\sqrt{{{\sigma }_{{\rm{r}}{\rm{e}}{\rm{a}}{\rm{l}}}}^{2}+{{\sigma }_{{\rm{s}}{\rm{h}}{\rm{u}}{\rm{f}}{\rm{f}}{\rm{l}}{\rm{e}}{\rm{d}}}}^{2}}}$$

where *µ* and $\sigma$ denote the mean and standard deviation, respectively. Neurons with *z*-scores exceeding 2.57 (corresponding to *P* < 0.01 in a two-tailed distribution) were categorized as spatially selective. To consider a possible directional tuning of a neuron on the maze, we restricted the analysis to the movement direction with a higher mean firing rate for each neuron.

Among cells categorized as spatially selective, we asked whether spatial tuning of these neurons also depends on the phase of navigation. To address this question, each navigation journey was discretized into eight equidistant positional fractions, and the firing rates at individual fractions or phases were assessed together with the absolute positions of the animalon the maze, by forming a firing rate matrix of phase and position (for example, Fig. 1e, Extended Data Fig. 3). To assess whether a neuron encodes phase and position conjunctively, the firing rate matrix was mean centred (the mean navigation-phase-dependent firing rate was subtracted from each column) and assessed for bias in firing rates relative to navigation phases. This bias was estimated by calculating the Frobenius norm of the mean centred matrix, which is defined as the square root of the sum of squared matrix elements. The statistical significance was assessed by calculating a distribution of Frobenius norms from 1,000 shuffled datasets among eight navigation phases. Neurons with the Frobenius norms exceeding the 95th percentile of the shuffled distribution were considered to encode position and navigation phase conjunctively.

### Two-dimensional firing rates and spatial information calculation

The arena (120 × 120 cm for OFC or 100 × 100 cm for CA1) was divided into 5 × 5-cm spatial bins, and the number of spikes and the overall time spent within individual bins during motion (>7.5 cm s^−1^) was calculated. The firing rate at each bin was estimated using an adaptive smoothing technique that optimizes the tradeoff between spatial resolution and sampling error^32^. In brief, for each spatial bin, an expanding circle was constructed until the following criterion was satisfied:

$$r > \frac{\alpha }{{n}_{{\rm{o}}{\rm{c}}{\rm{c}}}\sqrt{{n}_{{\rm{s}}{\rm{p}}{\rm{i}}{\rm{k}}{\rm{e}}{\rm{s}}}}}\,$$

where $r$ is the radius of the circle in bins, ${n}_{{\rm{o}}{\rm{c}}{\rm{c}}}$ is the number of samples occupied within the radius $r$, ${n}_{{\rm{s}}{\rm{p}}{\rm{i}}{\rm{k}}{\rm{e}}{\rm{s}}}$ is the number of spikes within the radius and $\alpha$ is a constant set to 200,000. Our positional sampling was interpolated to 1-ms resolution. Hence, ${n}_{{\rm{o}}{\rm{c}}{\rm{c}}}$ was the number of milliseconds the animal spent within a circle of radius *r* centred at the bin. Firing rate (spikes per second) in a given bin was calculated as 1,000 × ${n}_{{\rm{s}}{\rm{p}}{\rm{i}}{\rm{k}}{\rm{e}}{\rm{s}}}$/ ${n}_{{\rm{o}}{\rm{c}}{\rm{c}}}$. Spatial information for individual neurons in the OFC and CA1 was obtained from the rate maps using the following formula^52^:

$${\rm{S}}{\rm{I}}=\mathop{\sum }\limits_{i=1}^{N}{p}_{i}\frac{{\lambda }_{i}}{\lambda }{{\rm{l}}{\rm{o}}{\rm{g}}}_{2}\frac{{\lambda }_{i}}{\lambda }$$

where $N$ is the total number of spatial bins, ${p}_{i}$ is the probability of occupying the *i*th bin, ${\lambda }_{i}$ is the firing rate in the *i*th bin and $\lambda$ is the overall average firing rate of the neuron. The same formula was used to calculate spatial information of OFC neurons on the linear track.

### Well selectivity

The neuron’s selectivity for goal well was assessed based on its firing rates for each of 100-ms bins in the time range of −0.5 s to 2 s relative to motion onset of navigation, whereas the selectivity for the animal’s licking well (or current well) was assessed from its firing rates in the time range of −0.5 s to 2 s relative to the animal’s lick onset. To account for potential confounds of direction-specific firing, we used a two-way ANOVA with the well identity and the direction of the animal’s approach as two independent variables and the firing rate as a dependent measure. We used the ‘anovan’ function of MATLAB and used the type-II sum of squares for individual variables. Based on the *P* values for the well identity across all time points, we assessed the neuron’s selectivity to goal well and current well independently (a neuron can be categorized as both goal well and current well selective).

For the decoding analysis in Figs. 1, 2, we pre-selected neurons for a decoder based on a criterion of *P* < 0.05 at least in one of the time points in the range of −0.5 s to 2 s relative to the onset of either motion or licking. This procedure excluded neurons that were non-selective for the well identity, reducing the number of uninformative dimensions. For the visualization of neural activity trajectories in PCA-based reduced dimensions in Fig. 3b, we used a more stringent criterion of *P* values less than 0.01 over at least five consecutive time bins (500 ms) for the goal well selectivity.

Although the well selectivity was separately assessed for the current well or the goal well, we found that 83.03 ± 1.37% of the goal-well-selective neurons (by the criterion of *P* < 0.05) were also current well selective, and 69.38 ± 2.55% of the current-well-selective neurons were also goal well selective, suggesting overlaps of the two populations (Extended Data Fig. 3).

### Decoding analysis

We applied a decoder based on LDA that assigns individual class probabilities by setting class boundaries between multivariate Gaussian distributions fitted to data. In brief, a dataset from each recording session was divided into a training dataset and a test dataset, and a decoder was constructed from the training dataset by employing multiclass one-versus-one LDA using the ‘fitcecoc’ function of MATLAB with a regularization factor of 0.5 to reduce overfitting. We used uniform priors for all decoders. Next, we used the ‘predict’ function of MATLAB to obtain decoding probabilities of individual wells from the test dataset. This function uses an algorithm described by Hastie and Tibshirani^33^ to compute posterior probabilities from the pairwise conditional probabilities obtained using multiclass one-versus-one decoders. The trials during transition phases to new well combinations were excluded, and only correct trials were used for the decoder’s training. The unvisited wells in each session were excluded in the calculations of both decoding performance and its corresponding chance level. A population of neurons used for a respective decoding analysis for current well or goal well were pre-selected based on their well selectivity (using the method described in the previous section) because this procedure improved a decoder’s performance with better generalization to test data (Extended Data Fig. 7c, h), which is likely due to the reduction of unnecessary dimensions from uninformative neurons. For cross-validation of decoding performance, the training data of a decoder comprised all trials except the trial tested with the decoder as well as the one prior to this trial (that is, leave-two-out cross-validation). Additional details specific to each analysis are described in the following sections.

### Current well decoding

In the decoding analysis of the animal’s licking well (Fig. 1h, i, Extended Data Fig. 5), the data used for the training of a decoder comprised firing rate vectors of neurons (pre-selected based on their current well selectivity) at individual 100-ms bins in the range of −0.5 s to 3 s relative to lick onset, resulting in 36 rate vectors for the class label of licking well. This relatively long range of data (−0.5 s to 3 s) was chosen for a better generalization of well decoding over licking time (Extended Data Fig. 5j). Then, by using this decoder, we obtained the decoding probabilities of individual wells for all the 100-ms bins from −3 s to 6 s relative to lick onset (Fig. 1h, left) or from the beginning (motion onset) to the end (lick onset) of navigation (Fig. 1h, middle). For computing the decoding probability of the well that was run over by the animal, we restricted the analysis on trials when the animal’s running speed at the well exceeded 20 cm s^−1^ in a 500-ms window.

As a control analysis of decoding (Fig. 1h), we tested whether the well decoding depends on the direction of the animal’s approach (Fig. 1i, left). We trained a decoder from the data in which particular wells were approached only from one side of the linear maze and then tested the decoding performance when the animal approached the same wells from the other direction. We ensured that the decoder was trained with more than ten trials in which the target well was approached from one direction.

As another control analysis (Fig. 1i, right), we tested the possibility that the well decoding might depend on its paired wells in individual trial blocks. For this aim, we assessed the decoding performance of the wells when they are approached from newly paired wells. We trained a decoder with the data that excluded a trial block of a particular well combination but included the blocks in which the same wells were approached from other paired wells. We then tested the decoding performance of the wells approached from the pairs not used in the decoder’s training. The motivation behind this analysis is that, if the well identity is encoded by OFC neurons based on its spatial location, it should be decoded irrespective of its paired wells (or the animal’s start positions). The decoding was performed only when the target well was approached by the animal more than ten times in the training dataset.

### Goal well decoding

For the decoding of the animal’s goal well, we constructed a decoder based on the assumption that the goal well should be represented with the same pattern of neural activity between the beginning and the end of navigation (Fig. 2b). We thus trained the decoder from the data concatenated across two time ranges around motion onset and lick onset. We found that a dimensionality reduction procedure of the neural activity by PCA improved the subsequent decoding performance (Extended Data Fig. 7), likely because this decoding strategy entailed the construction of high-dimensional hyperplanes by concatenating two different time phases of the neural activity, and a dimensionality reduction procedure helped to constrain the hyperplane in a small number of crucial dimensions, thereby improving generalization of the decoder. Before implementing PCA, we used a soft-normalization technique described by Churchland et al.^34^ to adjust the range of firing rates across the neural population that were pre-selected based on their goal well selectivity (with the method described in the previous section). We then selected PCA dimensions that explain 85% of the data variance across the entire time duration of a recording session, obtaining the neural population activity in reduced dimensions. For each trial, vectors of the population activity in 100-ms bins were concatenated in the time range of −0.5 s to 0.5 s relative to motion onset, together with that of −0.5 s to 1 s relative to the subsequent lick onset at the destination, forming 27 vectors with the class label of goal well. These time ranges were chosen to capture the neural dynamics from the beginning to the end of navigation (Extended Data Fig. 7f).

The decoding was performed on the test dataset in the time range of either from −2 s to 2.5 s relative to motion onset (Fig. 2d, left) or from 1 s before motion onset to the subsequent lick onset at the goal well (Fig. 2d, right). The trials in which the goal wells were immediately adjacent to the animal’s current wells were excluded from the analysis.

### Chance level calculation

We tested a possibility that the goal well decoding could be explained by the neural activity encoding a task-relevant parameter other than the spatial position of goal well. We calculated five chance levels for goal well decoding, each of which corresponds to a specific null hypothesis (Extended Data Fig. 5).

We first tested the possibility that the goal well was not decoded based on its own identity. This possibility was tested by assessing the decoding performance when the well identities were exchanged by shuffling the class labels of training datasets.

We next asked whether the observed goal decoding can be explained by the animal’s running direction, speed, acceleration or trajectory length. To test these null hypotheses, we divided the training dataset into multiple groups. For testing the effect of running direction, we split the trials into two groups, each containing trials with the same running direction on the linear maze. Similarly, for testing the effect of trajectory distance, we divided the trials into groups of different trajectory lengths measured in terms of the number of wells between animal’s current and goal location; for testing the effect of running speed or acceleration, the trials were categorized into two groups (split across the median; analysis with quartile splits was also performed in Extended Data Fig. 5) according to the animal’s running speed or acceleration at motion onset. We then trained a decoder based on the training dataset with the class labels shuffled within individual groups. This procedure provides an estimate of how much well decoding can be possible with the neural activity difference resulting from a given behavioural parameter (without using precise well labelling for the decoder’s training), serving as an additional chance level.

The chance level calculation across all the sessions was implemented as follows. We first performed the decoding of all trials in a session using a decoder with shuffled class labels (as described above) and took the mean of decoding probability of the goal well. This process was repeated 100 times, resulting in a shuffled goal decoding distribution in each session. Examples of goal decoding from individual sessions and their corresponding chance levels (defined as 95th percentile of the corresponding shuffled distribution) are included in Extended Data Fig. 5. The subsequent computation of chance level across all the 18 sessions can intuitively be considered as a procedure to obtain a distribution of the means of 18 independent random variables. We randomly chose one sample from each of the 18 shuffled goal decoding distributions (with 100 samples each) and took their average, obtaining a representative of the session-averaged shuffled decoding probability of the goal well. This procedure was repeated 1,000 times to obtain a distribution of the means of shuffled goal decoding probability across the sessions. The chance level was set at the 95th percentile of the distribution.

The individual chance levels are depicted in Fig. 2f. To calculate the significance level of the decoding analysis in Fig. 2d, we used an aggregate chance level by taking the maximum of the five chance levels at each time point. For the decoding analysis of the animal’s licking well (Fig. 1h, i), we used only two null hypotheses by excluding the ones for the animal’s running speed, acceleration and trajectory length, as they are relevant only when the decoding includes a navigation phase.

### Supervised dimensionality reduction with LDA

LDA was applied for a dimensionality reduction procedure in Figs. 2b, 3c, 3e, f and Extended Data Fig. 9. In contrast to an LDA-based decoder that calculates class boundaries (described in the previous section), the LDA-based dimensionality reduction technique searches for a subspace onto which the projected data exhibit the best separation between categories. The detailed procedures of data matrix manipulations are described step-by-step as follows.

LDA is a supervised linear dimension reduction technique that computes a subspace with the maximum linear separability of data according to class labels. Formally, for C classes, LDA computes at most C − 1 eigenvectors corresponding to the eigenvalues of

$${({{S}}_{{\rm{w}}})}^{-1}{{S}}_{{\rm{b}}}$$

where ${{S}}_{{\rm{w}}}$ is the average within-class covariance matrix, and ${{S}}_{{\rm{b}}}$ is the covariance matrix of class means relative to the mean of all classes. Projecting the data on the subspace constructed by these eigenvectors results in the data with reduced dimensions by maintaining the maximum linear separability between classes. A subspace was calculated at each time point without concatenating the data over time for the analyses in Fig. 3c, e, f and Extended Data Fig. 9.

In the analysis in Fig. 2b, we projected the neural activity on the first LDA dimension (corresponding to the largest eigenvalue) to show the target-well-specific activity during the navigation. To find the common LDA dimensions across navigation, we used the neural activity data around the times of both motion and lick onsets of navigation (the same approach as the goal well decoding described in the previous section).

This procedure was also used to construct goal-well-specific neural trajectories by reducing goal-irrelevant activity (Fig. 3c, e, f, Extended Data Fig. 9). First, we carried out a general de-noising step by projecting neural activity to PCA dimensions that explain 85% of data variance (identical to the step described in the goal decoding section). Next, we applied the LDA-based dimensionality reduction procedure at individual time points of navigation. However, due to high-dimensional input data with a small sample number, LDA might overfit the subspaces resulting in poor generalization. We thus took two approaches to prevent this problem: regularization and cross-validation. For the regularization, we calculated the eigenvectors of the following matrix with a regularization factor:

$${({{S}}_{{\rm{w}}}+\lambda {I})}^{-1}\times {{S}}_{{\rm{b}}}$$

where ${I}$ is the identity matrix, and $\lambda$ is the regularization factor set to 1 (different values of $\lambda$ are tested in Extended Data Fig. 9). For the cross-validation procedure, we estimated LDA subspaces at individual time points of a particular trial from the training dataset excluding this trial (that is, leave-one-out cross-validation).

Because this procedure generated different subspaces (or axes) for individual trials, we projected the activity in the subspaces back to the original neural space common to all trials. For example, supposing that the data comprised *d*-dimensional neural data with *C* classes, the processed neural activity at a given time point of a trial was computed by using the following formula:

$${{\bf{x}}}_{{\rm{p}}{\rm{r}}{\rm{o}}{\rm{c}}}=({{\bf{x}}}_{{\rm{o}}{\rm{r}}{\rm{i}}{\rm{g}}}-{{\boldsymbol{\mu }}}_{{\rm{t}}{\rm{r}}{\rm{a}}{\rm{i}}{\rm{n}}})\times {M}{{M}}^{+}+{{\boldsymbol{\mu }}}_{{\rm{t}}{\rm{r}}{\rm{a}}{\rm{i}}{\rm{n}}}$$

where ${{\bf{x}}}_{{\rm{o}}{\rm{r}}{\rm{i}}{\rm{g}}}$ is a 1 × *d* vector of the original neural population activity, ${{\boldsymbol{\mu }}}_{{\rm{t}}{\rm{r}}{\rm{a}}{\rm{i}}{\rm{n}}}$ is a 1 × *d* vector of the mean neural activity of the training dataset, ${M}$ is a *d* × (*C*  1) matrix representing a transformation to the subspace computed by the regularized LDA based on the training dataset, ${{M}}^{+}$ is the pseudo inverse of ${M}$ and ${{\bf{x}}}_{{\rm{p}}{\rm{r}}{\rm{o}}{\rm{c}}}$ is a 1 × *d* vector of the processed neural activity. This entire procedure resulted in de-noising of neural signals according to LDA-based classification while maintaining the number of input dimensions (illustrated with examples in Extended Data Fig. 9).

### Linear modelling of neural dynamics

A regularized first-order linear dynamic model was used to simulate the neural activity dynamics during navigation (Fig. 3e, f). Modelling of a linear dynamic system can be considered a multiple linear regression problem in the following form:

$$\dot{{X}}={X}{A}$$

in which the matrix ${A}$ transforms the activity vector to the corresponding velocity vector. The regularized matrix ${A}$ can be obtained with the following calculation:

$${A}={({{X}}^{T}{X}+\mu {I})}^{-1}{{X}}^{T}\dot{{X}}$$

where ${X}$ is a data matrix with the activity at different times or trials in the row and the neuronal identities in the column, $\dot{{X}}$ is the time derivative of ${X}$, ${\boldsymbol{\mu }}$ is a regularization factor set to 5 (different values of ${\boldsymbol{\mu }}$ were tested in Extended Data Fig. 9) and ${I}$ is the identity matrix. For example, in the dataset with *p* trials, *T* time bins and *d* neurons, the matrix ${X}$ is created by concatenating all *p *× *T* data points, resulting in a *pT *× *d* matrix. Time-derivative components $\dot{{{\bf{x}}}_{t}}$ in the matrix $\dot{{X}}$ were computed as follows:

$$\dot{{{\bf{x}}}_{t}}={({\bf{x}}}_{t+1}-{{\bf{x}}}_{t-1})/2$$

where *x*_*t*_ _+_ _1_ and *x*_*t*_ _−_ _1_ are the activity vectors at the time step of *t *+ 1 and *t *− 1, respectively. The neural data used for model construction was pre-processed with the LDA-based de-noising approach described in the previous section. To account for non-linear neural trajectories with linear models, we fitted a linear dynamic model at every 500 ms of the neural data. Individual trajectories were simulated using the following equation in an iterative form:

$${{\bf{x}}}_{t}^{{\rm{s}}{\rm{i}}{\rm{m}}}={{\bf{x}}}_{t-1}^{{\rm{s}}{\rm{i}}{\rm{m}}}+{{\bf{x}}}_{t-1}^{{\rm{s}}{\rm{i}}{\rm{m}}}\times {A}$$

starting with the neural activity at motion onset:

$${{\bf{x}}}_{1}^{{\rm{s}}{\rm{i}}{\rm{m}}}={{\bf{x}}}_{{\rm{m}}{\rm{o}}{\rm{t}}{\rm{i}}{\rm{o}}{\rm{n}}\,{\rm{o}}{\rm{n}}{\rm{s}}{\rm{e}}{\rm{t}}}^{{\rm{d}}{\rm{a}}{\rm{t}}{\rm{a}}}+{{\bf{x}}}_{{\rm{m}}{\rm{o}}{\rm{t}}{\rm{i}}{\rm{o}}{\rm{n}}\,{\rm{o}}{\rm{n}}{\rm{s}}{\rm{e}}{\rm{t}}}^{{\rm{d}}{\rm{a}}{\rm{t}}{\rm{a}}}\times {A}$$

where ${{\bf{x}}}_{t}^{{\rm{s}}{\rm{i}}{\rm{m}}}$ is a simulated neural activity vector at time *t* (relative to motion onset), and ${{\bf{x}}}_{{\rm{m}}{\rm{o}}{\rm{t}}{\rm{i}}{\rm{o}}{\rm{n}}\,{\rm{o}}{\rm{n}}{\rm{s}}{\rm{e}}{\rm{t}}}^{{\rm{d}}{\rm{a}}{\rm{t}}{\rm{a}}}$ is the neural activity population vector at motion onset. We took a leave-one-out cross-validation strategy, in which all the parameters for modelling, de-noising and dimensionality reduction were obtained from the training dataset that excluded a test trial simulated by a model.

Goal decoding of the original and the simulated neural trajectories (Fig. 3f) was performed with the LDA-based decoding procedure described in the previous section, except that the decoders here were trained based on the de-noised neural activity from −0.5 s to 0.5 s relative to lick onset at the goal well. This narrow duration of 1 s was chosen to capture a snapshot of goal representation at lick onset without generalising over time.

### Statistical procedures

All statistical tests were two sided and non-parametric unless stated otherwise.

### Reporting summary

Further information on research design is available in the Nature Research Reporting Summary linked to this paper.

## Online content

Any methods, additional references, Nature Research reporting summaries, source data, extended data, supplementary information, acknowledgements, peer review information; details of author contributions and competing interests; and statements of data and code availability are available at 10.1038/s41586-021-04042-9.

## Supplementary information

Reporting Summary

## Data Availability

The datasets used for the figures can be obtained from the authors.

## Code availability

MATLAB codes for PCA, LDA (dimension reduction) and Isomap are part of the dimensionality reduction toolbox written by Laurens van der Maaten^35^. Other codes can be obtained from the authors.
